# Diverse types of expertise in facial recognition

**DOI:** 10.1038/s41598-023-28632-x

**Published:** 2023-07-14

**Authors:** Alice Towler, James D. Dunn, Sergio Castro Martínez, Reuben Moreton, Fredrick Eklöf, Arnout Ruifrok, Richard I. Kemp, David White

**Affiliations:** 1 School of Psychology, University of New South Wales, Sydney, 2052 Australia; 2 School of Psychology, The University of Queensland, Brisbane, 4072 Australia; 3 Sección Técnicas Identificativas, Comisaría General de Policía Científica, 28039 Madrid, Spain; 4 School of Psychology, The Open University, Milton Keynes, MK7 6AA UK; 5 Forensic Imaging Biometrics, Information Technology Section, National Forensic Centre, Swedish Police Authority, 581 94 Linköping, Sweden; 6 Forensic Biometrics, Netherlands Forensic Institute, 2497 GB The Hague, The Netherlands

**Keywords:** Psychology, Human behaviour

## Abstract

Facial recognition errors can jeopardize national security, criminal justice, public safety and civil rights. Here, we compare the most accurate humans and facial recognition technology in a detailed lab-based evaluation and international proficiency test for forensic scientists involving 27 forensic departments from 14 countries. We find striking cognitive and perceptual diversity between naturally skilled super-recognizers, trained forensic examiners and deep neural networks, despite them achieving equivalent accuracy. Clear differences emerged in super-recognizers’ and forensic examiners’ perceptual processing, errors, and response patterns: super-recognizers were fast, biased to respond ‘same person’ and misidentified people with extreme confidence, whereas forensic examiners were slow, unbiased and strategically avoided misidentification errors. Further, these human experts and deep neural networks disagreed on the similarity of faces, pointing to differences in their representations of faces. Our findings therefore reveal multiple types of facial recognition expertise, with each type lending itself to particular facial recognition roles in operational settings. Finally, we show that harnessing the diversity between individual experts provides a robust method of maximizing facial recognition accuracy. This can be achieved either via collaboration between experts in forensic laboratories, or most promisingly, by statistical fusion of match scores provided by different types of expert.

## Introduction

Facial recognition errors have far-reaching implications for public safety, civil rights, national security, and the criminal justice system. For example, security camera images are routinely used in criminal investigations to link suspects to crime scenes, and errors can lead to wrongful convictions. Further, photo identification is often used to control access to restricted spaces and goods, financial services, and to exercise civil rights, such as the right to vote. The average person makes between 20 and 30% errors when performing these types of facial image comparison tasks with unfamiliar faces (e.g., see ref.^1^), and many professional groups such as passport officers have shown similar error rates^2,3^.

Society’s increasing reliance on facial recognition means these errors are under greater scrutiny than ever before, and necessitates experts with proven accuracy in this challenging task. In recent years, three types of facial recognition ‘expert’—forensic examiners, super-recognizers, and Deep Neural Networks—have emerged independently from the fields of forensic science, psychology, and artificial intelligence, respectively. Here, we report the largest and most comprehensive comparison of these facial recognition experts to date.

In forensic science, practitioners known as facial forensic examiners identify persons of interest in police investigations and suspected cases of identity fraud in government identification procedures^4–7^. The forensic science of facial image comparison is part of the broader discipline of feature comparison methods that have received significant scientific scrutiny in recent years (e.g., see ref.^8^). A lack of evidence for the reliability of these methods led to calls from the National Academy of Sciences, influential US Government advisory committees, and others for validation studies and measurement of error rates^9–13^. In response to this call, recent studies have shown that forensic examiners outperform standard participant groups on unfamiliar face identification tasks (for a review, see ref.^4^). Evidence suggests forensic examiners acquire their expertise through professional training, deliberate practice and experience^4,14,15^.

Although the vast majority of people are error-prone when identifying unfamiliar faces, psychologists have identified a small proportion of the population—known as super-recognizers—who achieve extraordinary levels of accuracy without any specific training or experience^16,17^. Super-recognizers represent the upper tail of a continuum of natural variation in people’s ability to identify faces, which appears to be strongly heritable^18,19^. As a result, psychologists have argued that super-recognizers provide a route to high levels of accuracy in challenging real-world unfamiliar face identification tasks, and some police organizations recruit super-recognizers for face identification tasks^4,20–22^.

In artificial intelligence, facial recognition technology has seen remarkable advances in accuracy over recent years. The application of Deep Neural Networks (DNNs) to facial recognition has led to levels of accuracy that were unanticipated just 5 years ago^23–26^, meaning they too could play a role in improving forensic face identification. This success has also prompted psychologists and neuroscientists to evaluate DNNs as candidate models of face processing in the brain (e.g., see refs.^27–29^). This approach is plausible because neural networks were initially inspired by neurophysiology^30^ and their evolution continues to be shaped by discoveries in this field^31,32^. However, the extent of computational and representational similarity between DNNs and humans remains unclear, and only a small subset of available DNNs have been compared to humans (e.g., see refs.^27,28,33,34^).

A recent black box test run by the National Institute of Standards and Technology in the USA found forensic examiners, super-recognizers and DNNs achieve comparably high accuracy (83–96%; see ref.^23^). However, it is important to move beyond simple comparisons of accuracy to gain a deeper understanding of the basis of expertise in these three groups, and their relative strengths and weaknesses. We address this question here.

Given that forensic examiners, super-recognizers and DNNs appear to converge on equivalent high levels of accuracy^23^, it is possible that the perceptual and cognitive mechanisms driving their expertise also converge on similar computational solutions. The idea that expertise in face identification is homogeneous across different human observers is prevalent in psychological study of face recognition^35^, and consistent with broader concepts of convergent evolution^36^ and Ideal Observer Theory^37^. However, super-recognizers, forensic examiners and DNNs acquire their expertise via completely different means so there may be fundamental differences in the processes that support their face identification decisions^4,15^. We address these questions by characterising and comparing the perceptual and cognitive expertise of human face identification experts and state-of-the-art open-source facial recognition DNNs.

## Results

We first conducted detailed lab-based testing to benchmark 7 super-recognizers against published accuracy of forensic examiners and normative control groups on an extensive battery of face recognition tests, including professional tasks that mirror real-world forensic practice. We then entered 37 police and civilian super-recognizers into an extremely challenging international forensic proficiency test administered by the European Network of Forensic Science Institutes, and compared their performance on this test to 16 forensic examiners and 19 forensic laboratories from 27 forensic departments in 14 countries, and 10 DNNs. In addition to providing the most comprehensive comparison of accuracy between these types of experts, we provide a detailed comparison of the nature of the expertise underlying their high performance.

To summarize our key results reported below, we find striking differences between forensic examiners’, super-recognizers’ and DNNs’ facial recognition expertise, despite them achieving similarly high levels of accuracy. Super-recognizers’ expertise is characterised by fast decisions made with high confidence and a relatively strong response bias to say “same person”—which could lead to misidentification errors with catastrophic outcomes in forensic settings. Forensic examiners, on the other hand, make slow, careful decisions, show a neutral response bias, and strategically moderate their confidence ratings. DNNs show further divergence from both human expert groups in how they compute facial similarity.

Finally, we show that harnessing the diversity between these groups provides a robust method of maximizing facial recognition accuracy. We show this can be achieved either via collaboration in forensic laboratories, or by statistical fusion of responses by diverse types of facial recognition experts.

### Benchmarking super-recognizers against forensic examiners on lab-based tests

We initially recruited 7 super-recognizers for extensive lab-based testing based on their performance on an online version of the Glasgow Face Matching Test (GFMT; see ref.^38^) and their self-reported exceptional ability to recognise faces in their daily lives. To verify and assess their superior abilities, we then compared their performance to normative control data (*N*s = 54—290) on 5 standardised unfamiliar face identification tests. These tests included 2 face matching tasks (GFMT, Models), where participants saw two face images side-by-side and decided if they showed the same person or different people; 2 face memory tasks (CFMT + , CFMT-Aus) where participants were asked to learn and then recognise identities in increasingly challenging images; and, a general face identification test that is used to screen for super-recognition (UNSW Face Test), and involves a face memory task and a match-to-sample sorting task. Participants also completed 3 non-face object matching tests, which we compared to normative control data (*N*s = 48—1327), to investigate the extent to which their visual processing abilities were specific to human faces (Primate Faces, Fingerprints, MFFT). Full details of all tests and performance measures are provided in the Methods section, and individual scores and analyses are provided in supplementary materials.

Super-recognizers outperformed normative control scores by 2 standard deviations across all 5 unfamiliar face identification tests (Mean Cohen’s *d* = 2.97). Super-recognizers also outperformed normative control scores on the object matching tests (Mean Cohen’s *d* = 0.88) but to a lesser extent, suggesting that a substantial portion of super-recognizers’ expertise is face-specific (see Methods). Superiority in non-human primate face matching suggests that face recognition skills can generalise to some extent to morphologically similar object classes. However, superiority on fingerprint matching—a morphologically distinct class of visual pattern—indicates that domain general perceptual and/or cognitive matching abilities are enhanced in super-recognizers (for preliminary evidence of this generalisability see ref^23,39^).

Next, we compared the performance of super-recognizers to published forensic examiner and novice student control data on three face matching tests that reflect the type of face identification decisions made in real-world forensic practice: (i) Expertise in Facial Comparison Test (EFCT^40^), (ii) Person Identification Challenge Test (PICT^40^), and (iii) Facial Recognition Candidate List Test^41^. Major results from these ‘real-world’ inspired tests are described below.

#### Super-recognizers and forensic examiners show different perceptual processing in face identification

**Figure 1:**
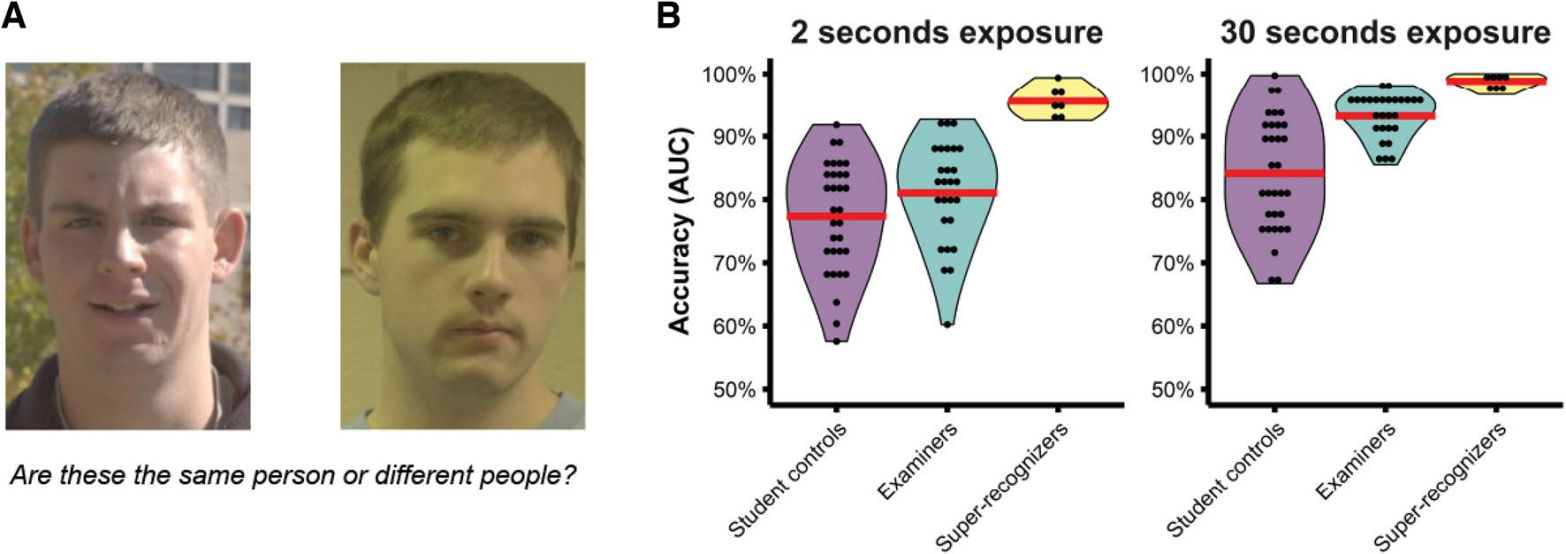
Different processing underlies face recognition expertise in super-recognizers and forensic examiners. (**A**): An example trial from the Expertise in Facial Comparison Test^40^ (EFCT). These images show different people. (**B**): Super-recognizers demonstrate superior accuracy after seeing face images for just 2 s, suggesting that fast, intuitive processes underlie their expertise whereas examiners’ expertise only becomes apparent when given sufficient time to deploy their slow, feature-by-feature comparison strategy. Violin plots show the distribution of performance for student controls, forensic examiners and super-recognizers on the upright conditions of the EFCT. Red lines show group means.

 We found evidence of different perceptual expertise between super-recognizers and forensic examiners on the EFCT^40^, a pairwise face matching task designed to reflect forensic facial image comparison where participants decide if two simultaneously presented faces show the same person or different people (see Fig. 1A).

Visual inspection of Fig. 1B reveals that super-recognizers in our study were more accurate on the EFCT than both student controls and forensic examiners in a previous study^40^. This super-recognizer advantage was especially striking when participants were given just 2 s to view the faces. In contrast, forensic examiners did not outperform student controls when given 2 s. They *only* outperformed student controls when given 30 s to view the faces. Super-recognizers can therefore achieve high levels of accuracy after viewing faces for a very short amount of time, whereas forensic examiners require more time to achieve the same level of accuracy. This finding points to differences in the perceptual processes underlying the expertise of super-recognizers and forensic examiners, and aligns with evidence that forensic examiners’ expertise is driven by a slow, feature-by-feature comparison strategy^42^.

#### Equivalent accuracy for super-recognizers and forensic examiners on professional face matching tasks

We tested the accuracy of our 7 super-recognizers on two professional face matching tasks that mirror real-world forensic face identification. The Person Identification Challenge Test^40,43^ is a difficult pairwise face matching task containing images that show face and body information, where participants must decide if the faces show the same person or different people (see Methods). Super-recognizers scored significantly higher than student controls (97% vs. 82%; *t*(36) = 5.35, *p* < 0.001, Cohen’s *d* = 1.79), but no different to forensic examiners (97% vs. 90%: *t*(31) = 1.55, *p* = 0.127, Cohen’s *d* = 1.26). The Facial Recognition Candidate List Test^41^ is designed to model the “1-to-many” task performed by passport issuance officers using facial recognition technology to screen for identity fraud (see Methods). On this test super-recognizers scored significantly higher than student controls (76% vs. 46%; *t*(53) = 5.32, *p* < 0.001, Cohen’s *d* = 2.27) but no different to forensic examiners (76% vs. 69%; *t*(13) = 1.03, *p* = 0.310, Cohen’s *d* = 0.47).

### International forensic proficiency test for face identification practitioners

Next, we sought to compare super-recognizers to the very highest global standards in forensic face identification. We therefore approached the European Network of Forensic Science Institutes (ENFSI) Digital Image Working Group (DIWG) who run an international industry proficiency test for forensic facial image comparison practitioners each year. Forensic science proficiency tests are designed to assess the abilities of forensic practitioners in realistic casework conditions to ensure they are performing at an acceptable level and to fulfil industry accreditation requirements. The annual ENFSI proficiency test therefore provides an ideal opportunity to compare the very best facial recognition solutions in challenging real-world conditions.

#### Forensic proficiency test participants

The 2018 ENFSI proficiency test reported here was developed by author SCM and administered to 16 forensic examiners and 19 forensic laboratories from 27 police, government, and private industry forensic departments across 14 countries in Europe, Africa, Oceania and the Middle East. Forensic examiners submitted individual decisions, and forensic laboratories submitted group decisions based on input from their laboratory team, which consisted of between 2 and 7 forensic examiners, super-recognizers and/or non-specialist practitioners. We then administered the proficiency test to super-recognizers, facial recognition DNNs, and novices.

Thirty-seven super-recognizers completed the proficiency test. Six were the top-performing super-recognizers described above. Seven were recruited from a police super-recognizer unit which was established following extensive performance testing by another research group. The remaining 24 super-recognizers were recruited by screening the face recognition abilities of 1600 people using three standardised online tests (UNSW Face Test, GFMT, CFMT +). Participants who scored 2 SDs above the normative control mean on all three tests—an extremely strict inclusion criteria for super-recognition—were invited to participate. A detailed description of super-recognizer recruitment is provided in the Methods section.

One-hundred and six novice controls completed the proficiency test. Sixty-five were police officers from London’s Metropolitan Police Service with no professional experience in facial image comparison. The remaining 41 novices were undergraduate students from UNSW Sydney. Finally, we compared performance of these groups to 10 recent open-source face recognition DNNs that achieved state-of-the art performance (see Methods for details of the DNNs). The final participant sample therefore consisted of 16 forensic examiners, 19 forensic laboratories, 37 super-recognizers, 10 DNNs and 106 novices.

#### Forensic proficiency test procedure

**Figure 2:**
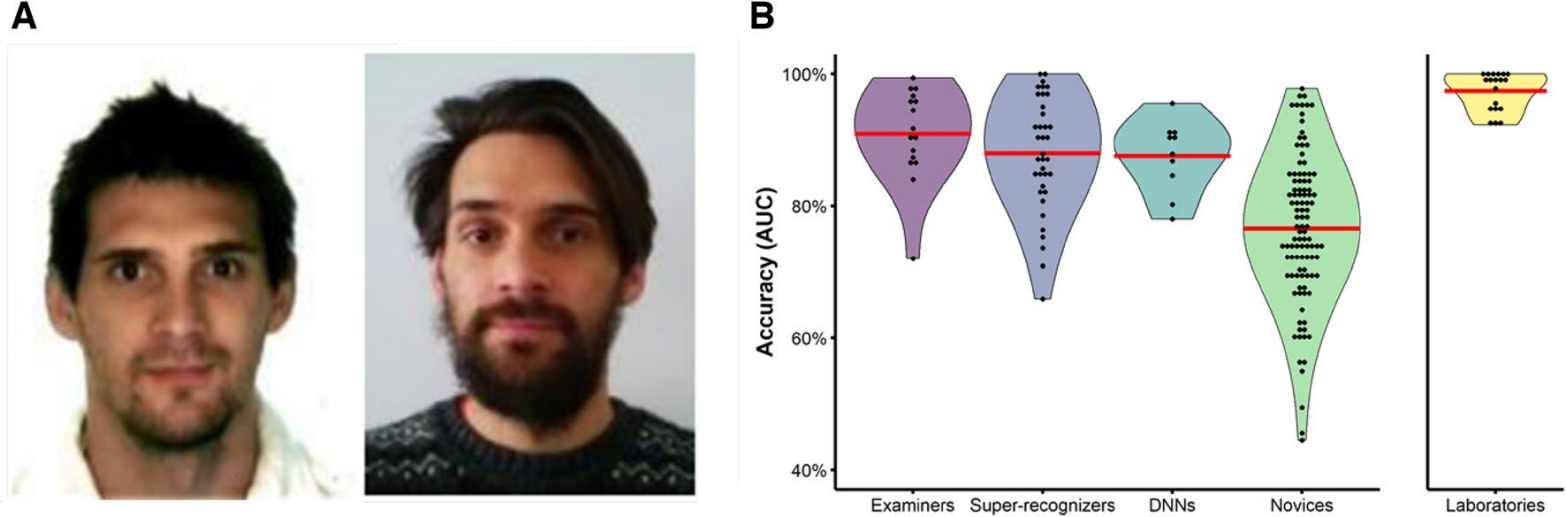
Comparison of the best available face recognition solutions. (**A**) Example 1-to-1 comparison from the 2018 ENFSI proficiency test. These images show the same person. (**B**) Ranked accuracy of the best available face recognition solutions. Red lines show group means. Forensic examiners, super-recognizers and DNNs achieved equivalent levels of accuracy and were all superior to novice participants. Group-based laboratory decisions (right) were more accurate than decisions reached by individuals, pointing to benefits of collective decision-making.

The proficiency test consisted of 20 challenging 1-to-1 face comparisons (13 same person, 7 different people) representative of high-quality forensic casework (see Fig. 2A). All participants responded using an 11-point scale from − 5 (*Extremely strong support different people*) to 5 (*Extremely strong support same person*), where the midpoint 0 indicates the comparison provides “inconclusive” evidence for either conclusion. Note that we have shortened these response scale labels for brevity (see supplementary materials for verbatim wording).

Forensic examiners, forensic laboratories and 19 super-recognizers were sent the raw image files and asked to return the completed test within 2 months. This meant the forensic practitioners could use their organisations’ standard operating procedures, tools, and software to complete the test, providing a realistic test of their abilities in operational settings. The remaining 18 super-recognizers and all control subjects completed the test online, where they could change their answers and navigate back and forth between comparisons, just as the other participants could. We verified that the results reported below are robust to the online/offline procedural differences between groups (see supplementary materials).

#### Accuracy of super-recognizers, forensic examiners, DNNs and forensic laboratories on the international forensic proficiency test

To enable comparison between human and DNN decisions we calculated accuracy on the proficiency test for each human participant and DNN using Area Under the ROC Curve (AUC). The accuracy of each group is shown in Fig. 2B, ranked from highest to lowest by mean group performance. The accuracy of group decisions made by forensic laboratories is presented alongside the accuracy of individuals for comparison. Full details of the following one-way ANOVA, follow-up, and completion time analyses are provided in supplementary materials.

All expert groups significantly outperformed novices (76.2%; *ps* ≤ 0.004, Cohen’s *ds* ≥ 0.99), with forensic examiners (91.0%), super-recognizers (88.0%) and facial recognition DNNs (87.6%) performing the test equally well (*ps* ≥ 0.210, Cohen’s *ds* ≤ 0.52). Consistent with the detailed lab-based testing described earlier forensic examiners reported taking much longer (mean: 8.7 h) to complete the 20-item test than super-recognizers (mean: 1.1 h) despite achieving equivalent accuracy, once again pointing to differences in the perceptual processes underlying their expertise.

Interestingly, forensic laboratories significantly outperformed all other groups, achieving 97.4% accuracy (*ps* < 0.001, Cohen’s *d* ≥ 1.25). This result indicates that group decision making in forensic laboratories results in highly accurate face identification decisions. Although we know forensic examiners, super-recognizers and non-specialist practitioners contributed to these decisions, we do not know how each group arrived at their decisions, or what decision-making tools they used, to make inferences about the source of the forensic laboratory advantage. We therefore examine the basis of the superiority of collective face identification decisions like those made in forensic laboratories by running a fusion analysis at the end of this section.

#### Super-recognizers and forensic examiners make different errors and responses across the response scale

**Figure 3:**
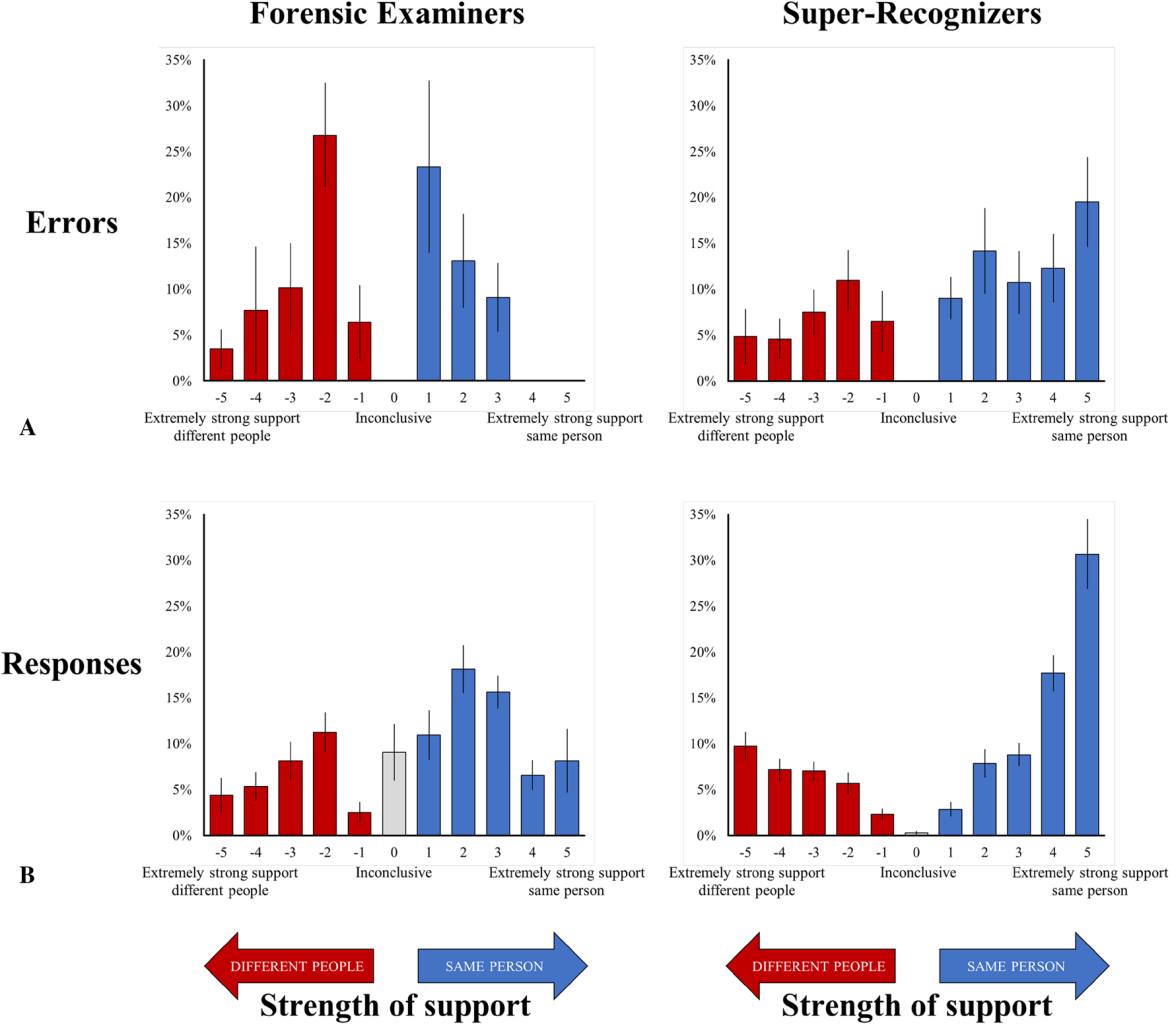
Forensic examiners avoid costly errors made by super-recognizers. (**A**): A large proportion of super-recognizers’ errors were high confidence ‘same person’ errors (responses of 4 and 5). Forensic examiners never made these errors. In forensic settings, false positive errors of this sort may lead to wrongful convictions, especially when made with high confidence. (**B**): Unlike forensic examiners, super-recognizers tended towards high confidence responses. This tendency was most apparent for ‘same person’ decisions, reflecting a response bias for super-recognizers to respond ‘same person’. Also, nearly 10% of forensic examiners’ responses and almost none of super-recognizers’ responses (0.27%) were ‘inconclusive’. Error bars show standard error of the mean. See supplementary materials for an extended version of this figure including novices.

In real-world forensic settings, face identification decisions are typically provided on response scales that specify decision confidence. These levels of confidence can have a large impact on how evidence is used and interpreted in police investigations and criminal trials. We therefore compared the way forensic examiners and super-recognizers distribute their decision confidence on a standard 11-point response scale used in forensic casework, by examining their errors and responses across the response scale. Results are shown in Fig. 3 and full details of analyses are provided in supplementary materials.

We found striking differences between forensic examiners’ and super-recognizers’ errors across the response scale (see Fig. 3A). The majority of forensic examiners’ errors lie towards the middle of the response scale, as evident by the inverted U-shaped distribution in Fig. 3A. Because the middle of the response scale indicates the lowest levels of confidence, this pattern of errors suggests forensic examiners’ responses are calibrated to their accuracy—that is, they expressed low confidence when they made errors. In contrast, super-recognizers do not show evidence of this calibration. Instead, the distribution of super-recognizers’ errors is skewed towards making misidentification errors, i.e., incorrectly declaring images of different people as the same person.

It is particularly concerning that nearly one third (31.8%) of super-recognizers’ errors were high-confidence ‘same person’ errors (responses of 4 or 5). High-confidence errors can have profound consequences in the criminal justice system because if presented as evidence in court they would be highly persuasive to judges and jurors, and could lead to wrongful convictions. Critically, forensic examiners did not make a single error of this kind. This finding suggests forensic examiners—but not super-recognizers—deliberately adopt a decision-making strategy that is better calibrated to their accuracy and protects against misidentification errors.

We also found striking differences in how forensic examiners and super-recognizers used the response scale (see Fig. 3B). Super-recognizers used the extreme ends of the response scale (− 5, 4, 5) significantly more, and the midpoints of the scale (− 2, 0, 1, 2, 3) significantly less, than forensic examiners (*ps* ≤ 0.017, see supplementary materials for details of these Mann–Whitney U analyses). Notably, forensic examiners often responded “inconclusive” (0), whereas super-recognizers almost never did (9.1% vs. 0.3%). Forensic examiners were also more likely to respond “inconclusive” on comparisons that carried a greater chance of error for super-recognizers, suggesting they strategically respond “inconclusive” on those comparisons to avoid making the errors super-recognizers make (see supplementary materials; c.f., ref.^7^).

Differences in response scale use were borne out in response criterion. Super-recognizers had a strong and significant response bias to respond “same person” (*M* = − 1.08; *t*(36) = 3.98, *p* < 0.001, Cohen’s *d* = 0.65), whereas forensic examiners had a neutral response bias, meaning they were no more likely to respond “same person” than “different people” (*M* = − 0.10; *t*(15) = 0.22, *p* = 0.828, Cohen’s *d* = 0.06). Detailed criterion analyses are provided in supplementary materials.

#### Humans and facial recognition DNNs assess facial similarity differently

**Figure 4:**
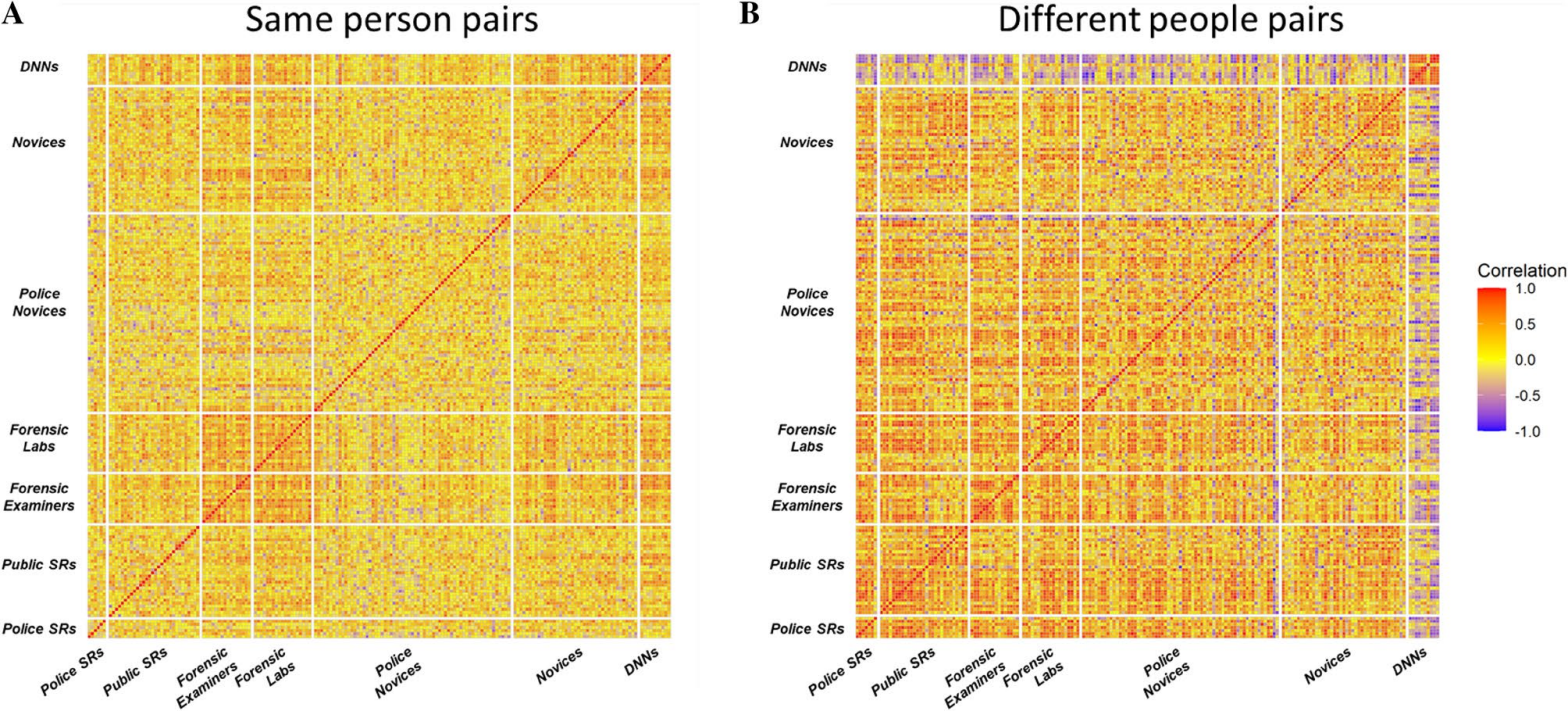
Correlation heatmaps of the similarity of responses between 177 human participants and 10 facial recognition DNNs. Red pixels indicate a positive Spearman’s rank-order correlation, blue pixels indicate a negative correlation, and yellow pixels indicate zero correlation. While humans and DNNs tended to agree on the similarity of same-person face pairs (**A**), they showed striking disagreement on the similarity of different-people face pairs (**B**), indicated by the increase in the number of blue pixels visible on the top and right-hand edge of the heatmap. The heatmaps were generated using the ggcorrplot package^44^ in R.

Next, we sought to compare humans’ representations of facial similarity to that of facial recognition DNNs. Figure 4 shows the Spearman’s correlations between the 20-item test responses for every participant in the dataset (177 humans and 10 DNNs). Note that we excluded one super-recognizer from the “same person” pairs analysis and another from the “different people” pairs analysis because they only made one response for that trial type. More red saturation of pixels indicates stronger agreement as to which faces look most similar to one another, and more blue saturation indicates stronger disagreement.

For “same person” pairs (Fig. 4A), there was agreement between DNN and human responses (average ρ = 0.27). We observed a similar pattern of agreement between DNNs and super-recognizers (average ρ = 0.26) and DNNs and forensic examiners (average ρ = 0.40).

For “different people” pairs (Fig. 4B) however, we found striking disagreement between DNN and human responses (average ρ = − 0.19), as indicated by the blue regions along the top and right-hand edge of Fig. 4B. We observed a similar pattern of disagreement between DNNs and super-recognizers (average ρ = − 0.22) and DNNs and forensic examiners (average ρ = − 0.21). These findings mean that for photos of different people, the more dissimilar they look to humans, the more similar they look to DNNs. This pattern points to profound differences in the way that humans and the DNNs we tested compute facial similarity.

Figure 4 also allows us to examine the agreement in responses *between* and *within* different groups of humans and DNNs. We note that despite differences in super-recognizers’ and forensic examiners’ perceptual processing and use of the response scale described above, they show agreement in their responses to same person (average ρ = 0.25) and different people pairs (average ρ = 0.37). Human experts therefore arrive at a similar rank-ordering of facial similarity, indicating they converge on similar representations of facial similarity. Additional comparisons are reported in supplementary materials.

#### Optimising accuracy by combining diverse responses of super-recognizers, forensic examiners and DNNs

In the final analysis, we examined the benefits of statistically combining or ‘fusing’ independent responses from small groups of face identification experts. Fusion serves to reduce subjectivity and produce more accurate decisions than individuals^45,46^. The purpose of this fusion analysis is threefold. First, combining the decisions of multiple human experts provides a model of forensic laboratories, allowing us to investigate the source of their superior accuracy described earlier (see Fig. 2B). Second, research in other domains shows fusion effects are strongest when there is greater diversity in the decision-making strategies employed by group members (e.g., see ref.^47^). A fusion analysis can therefore provide converging evidence of the diversity in cognitive processing by different types of expert. Third, harnessing the diversity between expert groups via fusion could provide a means to optimise facial recognition accuracy, beyond that achievable by an expert group alone.

**Figure 5:**
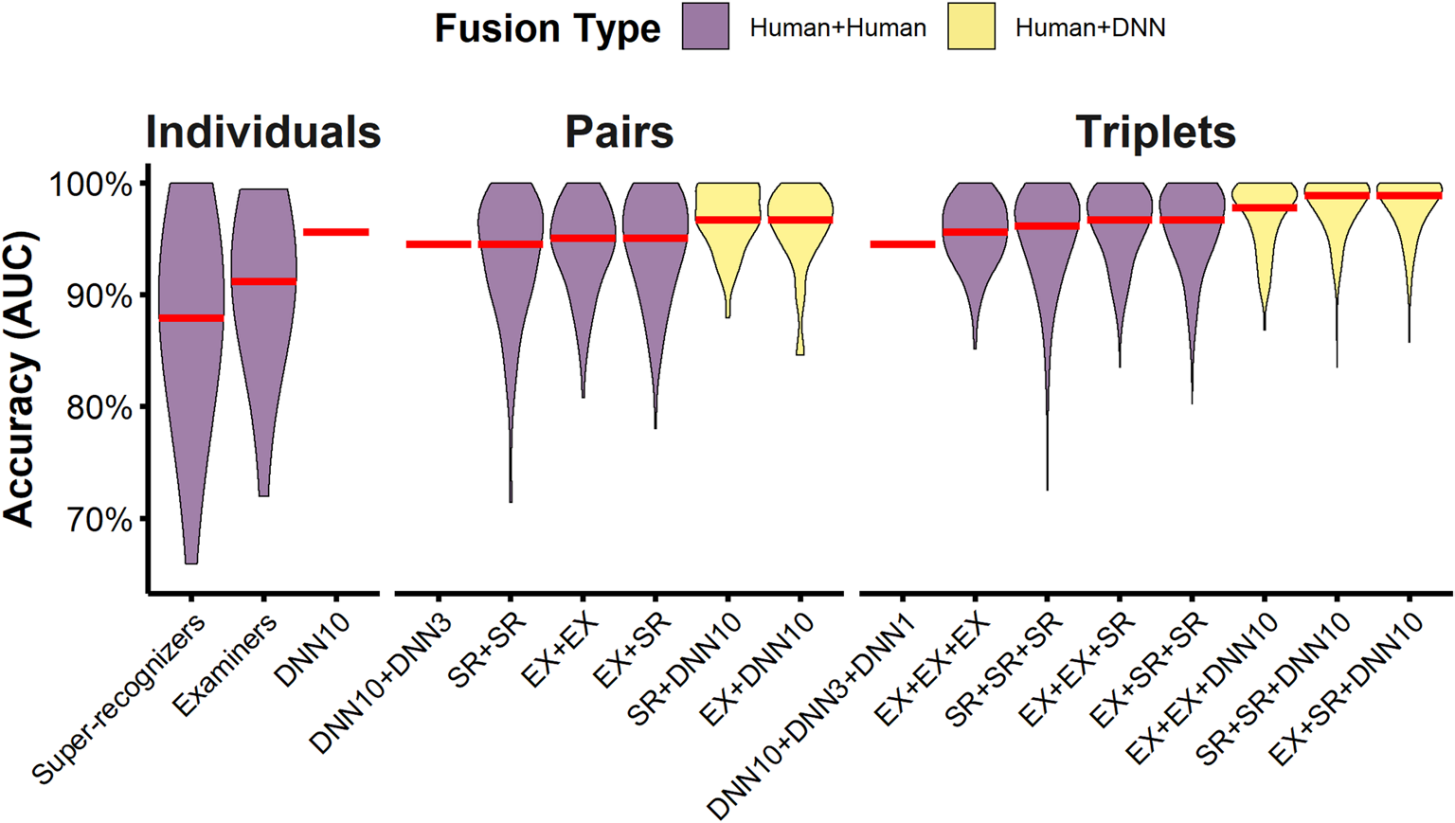
Optimal accuracy in face recognition is achieved by aggregating responses of diverse experts. Violin plots show the distribution of accuracy scores (AUCs) for each fusion. Red lines show median accuracy. Comparison of individuals, pairs and triplets shows increased accuracy with increasing group size. The best results occur when fusing responses from both humans and DNNs (yellow), resulting in more accurate decisions compared to either human–human (purple) or DNN-DNN fusions. Fusing human experts’ decisions models decisions made by forensic laboratories, and the benefits of doing so may explain the superiority of forensic laboratory decisions. Here, we report the best performing DNN (DNN10) for the individuals analysis, and the DNNs that produce the strongest fusion effects with DNN10 for the pairs (DNN3) and triplets (DNN3 and DNN1) analyses. However, we note the results are consistent for almost all DNNS. SR = super-recognizer; EX = forensic examiner.

To conduct the fusion analysis, we randomly constructed nominal groups of either 2 or 3 individual human experts (super-recognizers, forensic examiners), the 2 or 3 highest performing DNNs, or a mix of human experts and DNNs. We then computed average responses of each group to each image pair, and then calculated the accuracy of the collective decisions made by the group. The results of the decision fusion analysis are shown in Fig. 5.

In general, groups—consisting of humans and/or DNNs—showed improvements in accuracy relative to individual decisions, consistent with previous work^23,45,48^ (see supplementary materials for full analysis).These fusion effects may explain the superiority of forensic laboratory decisions described earlier (see Fig. 2B). We know that laboratory groups were comprised of an average of 2.9 (range 2 to 7) forensic examiners, super-recognizers and/or other staff. Here, aggregating the independent decisions of 2 or 3 forensic examiners, super-recognizers or a mix of both these groups achieved similarly high levels of accuracy as forensic laboratories.

Most importantly, human-DNN fusions almost always outperformed human–human fusions (see also ref.^23^), and DNN-DNN fusions (see yellow violins in Fig. 5; see supplementary materials). Human and DNN fusions achieved the very highest levels of accuracy on this extremely challenging forensic proficiency test (AUC range: 92% – 99%; see supplementary materials). This finding therefore provides further evidence that different representations underlie human expert and DNN face identification decisions, and reveals that harnessing the diverse expertise of humans and DNNs provides the best available solution to maximising facial recognition accuracy.

## Discussion

Three expert solutions to face recognition have emerged in recent years: forensic examiners, super-recognizers, and Deep Neural Networks. Here, we provided a comprehensive comparison of the accuracy and responding behaviour of these expert groups, using an extensive battery of lab-based face identification tasks and an international forensic proficiency test involving staff from 27 forensic departments in 14 countries. We found strong evidence that super-recognizers, forensic examiners and facial recognition DNNs have different expertise in face identification. Super-recognizers and forensic examiners differed both in terms of perceptual processing strategies and how they mapped their perceptual confidence onto the response scale. DNNs also diverged from humans in their representations of facial similarity. These findings provide novel insights into the nature of face recognition expertise, and inform how and when different face recognition experts should be deployed in real-world practice.

Super-recognizers and forensic examiners used different perceptual strategies to extract identity information from faces, despite achieving equivalent accuracy. Super-recognizers extracted maximal identity information within 2 s, whereas forensic examiners required up to 30 s. This pattern is consistent with our recent proposal that super-recognizers identify unfamiliar faces by exploiting the fast, automatic neural pathways evolved to recognize familiar faces, and that forensic examiners bypass this “normal” face recognition system by deliberately employing a slow, feature-by-feature comparison strategy^4,5,15,42^. Notably, these contrasting forms of expertise reflect key debates in the literature on the role of holistic versus featural processing in face recognition expertise^15,49^, and the role of System 1 versus System 2 processing in expert performance more broadly^50^.

Although super-recognizers and forensic examiners converged on similar rank-ordering of face similarity, they diverged in how they mapped these representations of facial similarity onto the response scale. This produced a striking tendency for super-recognizers to express high levels of confidence when making critical ‘same person’ errors. In contrast, forensic examiners appeared to use the response scale in a deliberate and strategic manner to protect against these misidentification errors. Forensic examiners’ ‘strategic conservatism’ may be due to forensic practitioners’ heightened awareness that these types of errors can lead to wrongful convictions in professional practice. However, we note that the police super-recognizers in our sample had a stronger response bias to say “same person” than the super-recognizers recruited from the general public. This indicates that simply making identification decisions in a forensic organization does not produce strategic conservatism in itself, pointing to the importance of training and experience in moderating response confidence (see supplementary materials). Given the increasing trend for super-recognizers to make face identification decisions in forensic settings, this result also highlights the need for organizations to carefully consider which face identification roles super-recognizers are suitable for^21,22,51–54^, and for researchers to establish whether super-recognizers can be trained to be more conservative.

Whereas human experts converged on similar rank-ordering of face similarity, DNNs and humans showed considerable disagreement regarding the similarity of photos of different people. This points to different representational geometry underlying the ‘face space’ of state-of-the-art DNNs and the most accurate human observers (see refs.^29,33,55^). Recent work has shown relatively high agreement between human judgments of facial similarity and one of the DNNs we tested (DNN1: VGG16), but the study used computer generated images of very dissimilar looking faces^33^. Here, we show important divergence in face similarity judgments on a range of DNNs using more challenging and naturalistic face images.

This divergence between humans and DNNs has implications for the use of modern facial recognition DNNs as cognitive models of the human face processing system (e.g., see refs.^27,36^). It is notable that humans’ face processing systems have likely evolved to serve different functions to the unfamiliar face matching that participants performed here. Indeed, given that the face matching task only became possible after the invention of photography some 200 years ago, any ‘natural’ human expertise on this task is likely to be a by-product of other evolutionary pressures, for example to recognize the faces of people we know under a variety of conditions and to read their emotional expressions. In contrast, DNNs have been engineered for the specific purpose of deciding whether images of unfamiliar faces are the same person. It is possible that these differences are at the source of representational differences, and so future work in modelling human face recognition could explore ways to engineer DNNs using constraints inspired by the ecology of human face processing (e.g., see refs.^56,57^).

Aggregating human expert and DNN responses produced larger fusion effects than aggregating the decisions of either group alone (see also ref.^23^). We conclude that this advantage arises from combining divergent representations of face identity in humans and DNNs. Given this apparent benefit of diverse cognitive processing, it is interesting to note that modern facial recognition DNNs share similar architecture and are trained on similar image datasets with similar training procedures (see Table 1 in the Methods section). This is likely to explain why we did not observe large fusion benefits when combining the responses of different DNNs and suggests that greater benefits of DNN fusion could be achieved by increasing the diversity between systems, for example by systematically diversifying their training and development. In support of this, DNN8 (ArcFace) had relatively low correlations with the other DNNs and yet showed the strongest fusion effects when combined with the other DNNs (see supplementary materials).

The diversity of cognitive processing by humans and DNNs also has important implications for the growing movement to explain AI to humans so they can catch AI errors (i.e., ‘explainable AI’; see^58–60^), particularly given that legislation, public opinion, and practicalities often demand human oversight of algorithm decisions^62^. The success of explainable AI is likely to be limited by the extent to which humans and AI make different errors, otherwise humans will make the same errors as the AI and fail to appropriately safeguard the system. This is yet another reason why a goal of this field should not be to make humans process faces like DNNs do, but rather to deliberately engineer diversity so that it can be harnessed in collective decisions.

We found that forensic examiners, super-recognizers and DNNs all achieve high accuracy, but each have distinct strengths and weaknesses which make them suited to different real-world face identification roles. Super-recognizers can make decisions quickly and rarely miss targets. Super-recognizers are therefore most suited to time-critical roles where the priority is to avoid false negative errors (misses) in the interests of public safety, such as border control, surveillance, searching for a face in a crowd, and reviewing the output of automated database searches. In these roles, false positive errors—which super-recognizers are prone to making—can often be eliminated quickly by further investigation. However, super-recognizers’ propensity for high confidence errors makes them ill-suited to high-stakes roles where the consequences of error might be permanent or life-changing, such as providing evidence to the court or assessing asylum eligibility, and where high confidence may cause judges and jurors to place undue weight on the evidence.

In contrast, forensic examiners are ideally-suited to making high-stakes identification decisions because they strategically avoid errors, know when *not* to make a decision (i.e., respond “inconclusive”; see refs.^63,64^), and adopt a neutral response bias. Further, their feature-by-feature comparison strategy (see refs.^5,42^) and well-calibrated confidence estimates lend themselves to legal requirements for expert witnesses to explain decision-making procedures and communicate the strength of their evidence in court.

In many applied security and forensic settings, face recognition decisions are often the output of complex systems that include different groups of human experts and DNNs^65,66^. For example, the Federal Bureau of Investigation in the United States provides a facial recognition service for law enforcement that involves automated facial recognition searches with review by human operators^67^. The results we have presented here call for careful consideration of the chains of decision making that are deployed in these systems, and the way that different types of experts—human and DNNs—are used within them. For instance, an evidence-based policing system might employ super-recognizers to screen automated “1-to-many” mugshot database search results for an offender. Suspected matches could be escalated to forensic examiners—or better yet, a forensic laboratory—who conduct an independent examination and then corroborate decisions using a second DNN. To quantify the benefits of this approach, future research could look to form diverse teams of humans and DNNs, and then test the system’s overall performance on a new set of facial comparisons.

In conclusion, there are multiple routes to expertise in facial recognition, and this contrasts with prevailing theory and practice in this interdisciplinary field. Psychological work has typically treated this expertise as a unitary construct, and has aimed to characterise the common aspects that distinguish it from other types of visual processing (e.g., see refs.^68–70^, but c.f. ref.^35^). Likewise, studies of individual differences in people’s expertise with faces have been guided by the goal of identifying a common skill that determines a person’s ability in the task (e.g., see ref.^69^). In forensic science, there is a strong emphasis on training individual experts to arrive at the same decisions (i.e., to “harmonize” their responses; e.g., see ref.^9^). In artificial intelligence, the vast majority of DNNs are developed using similar engineering approaches and are trained on similar face databases (but see refs.^57,70^).

In contrast, the work we have presented here raises the possibility that there is no single solution to the problem of accurate facial recognition, and that there are instead a range of near-perfect approaches. Optimal accuracy can only be achieved by aggregates of these approaches. Intelligent design of facial recognition systems should therefore not prioritise one type of expertise over another, but rather harness the diversity in cognitive processing between humans and DNNs to build more robust facial recognition systems.

## Methods

### Benchmarking super-recognizers against forensic examiners on lab-based tests

#### Participants

We tested 7 super-recognizers: DP a 31-year-old male, TI a 37-year-old male, DB a 27-year-old female, HC a 48-year-old male, CM a 24-year-old female, YS a 25-year-old female and CT a 46-year-old male. These super-recognizers originally approached our lab because they believed they had superior face identification abilities and were invited to participate in the lab-based assessment if they scored 95% or higher on the Glasgow Face Matching Test^38^.

This research was approved by the UNSW Human Research Ethics Advisory Panel and informed consent was obtained from all subjects in the study. All methods were performed in accordance with relevant guidelines and regulations. The identifiable face images in this paper were sourced from publicly available image sets or published with the informed consent of the subject.

#### Materials

##### Face matching tasks

**Figure 6:**
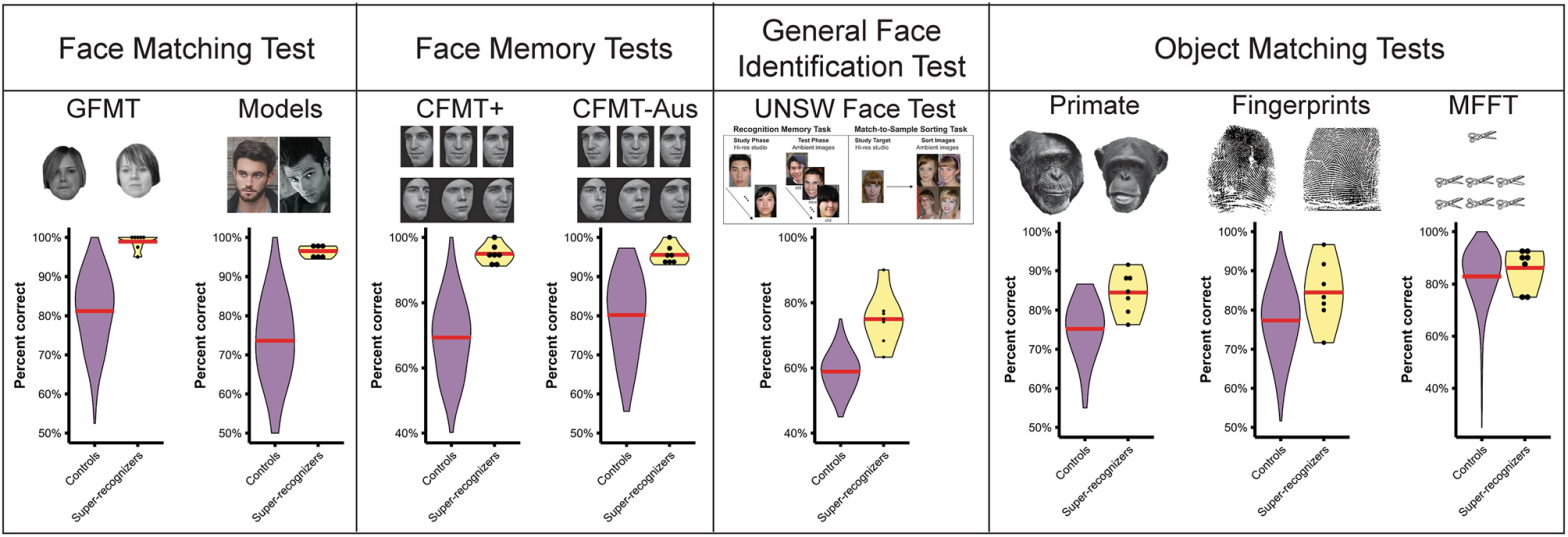
Super-recognizers show consistent and face-specific identification expertise. Violin plots show the distribution of performance for super-recognizers and controls on the test battery. Red lines show group means. Super-recognizers outperformed controls on a battery of standardised tests measuring face matching (GFMT^38^, Models^71^), face recognition memory (CFMT + ^78^, CFMT-Aus ^73^), and general face identification abilities (UNSW Face Test^74^). To a lesser extent, they were also better than controls on both the Primate Matching Test and Fingerprint Matching Test but not the MFFT test, suggesting some overlapping ability across domains in perceptual matching. We are unable to show the primate and fingerprint stimuli used in the test. Example primate faces were obtained from Pixabay (https://pixabay.com/images/search/monkey%20face/) and are released under the Pixabay License. Fingerprint images are by Metrónomo and licenced under SelfCC BY-SA 2.5 AR (https://creativecommons.org/licenses/by-sa/2.5/ar/deed.en).

*Glasgow Face Matching Test (GFMT) Short Version.* The GFMT^38^ (see Fig. 6) is a standard measure of face matching ability. In the short version, participants decide whether 40 face pairs (20 match, 20 non-match) depict the same person or two different people. Both images were captured on the same day, minutes apart, but with different cameras. Normative control data from 194 participants were sourced from Burton, et al.^38^.

* Models Face Matching Test (Models)*. The Models Face Matching Test^22,71^ (see Fig. 6) was originally designed to assess the face matching abilities of members of the London Metropolitan Police’s super-recognizer unit. The images in this test are professional images of multiple male models with various clothing, hairstyles, lighting conditions and are taken with different cameras. These images were shown in full colour and were cropped so that only the face was visible. The test required participants to make ‘same person’ or ‘different people’ identity decisions for 90 pairs of faces (45 match, 45 non-match). Normative control data from 54 participants were sourced from Robertson, et al.^22^.

##### Face memory tasks

*Cambridge Face Memory Test Long Form (CFMT* +*).* The CFMT + ^17^ (see Fig. 6) measures participants’ ability to learn and recognize unfamiliar faces. Participants learn each target face from different viewpoints and then attempt to recognize the six targets in a three-alternative-forced-choice test. The test contains four stages that successively increase the difficulty of the task by introducing either untrained views and lighting conditions of the images or by adding visual noise to the images. Normative control data from 254 participants were sourced from Bobak, et al.^72^.

*Cambridge Face Memory Test Australian (CFMT-Aus).* The CFMT‐Aus^73^ (see Fig. 6) is the Australian version of the standard CFMT paradigm and uses images of Australians that match the ethnicity of most Australian participants (i.e. Caucasians with British‐heritage). This test follows the same design as the CFMT + but without the fourth stage. Normative control data from 75 participants were sourced from McKone, et al.^73^.

##### General face identification test

*UNSW Face Test.* The UNSW Face Test^74^ (see Fig. 6) is a new test of face recognition designed to discriminate between participants who typically achieve ceiling performance on other standardized tests of face identification like the CFMT + and GFMT. This test uses a mixture of studio-captured images and ambient images taken from social media (i.e. Facebook) of each target. The UNSW Face Test consists of two tasks completed in a fixed order: a recognition memory task and a match-to-sample sorting task. In the recognition memory task, participants memorize 20 faces, shown for 5 s each before being tested on a new image of each of the same identities (20 total) intermixed with 20 foil faces. In the match-to-sample task, participants memorise a target face for 5 s before sorting a ‘pile’ of four ambient images by dragging an image to the right if it shows the target or to the left if it does not. Participants are told the pile could contain between 0 and 4 images of the target. The remaining images in the set of 4 are of the target’s foil. For this task participants complete two practice trials, followed by 20 trials in a fixed order. Normative control data from 290 participants were sourced from Dunn, et al.^74^.

##### Object matching tests

*Primate Face Matching Test.* We designed this test to mirror the task conditions of the GFMT but with non-human faces (see Fig. 6). Participants saw 59 pairs of images consisting of chimpanzee (Pan troglodytes) and rhesus monkey (Macaca mulatta) faces, and decided if they depicted the same individual or different individuals. Thirty of the image pairs showed the same individual and 29 image pairs showed faces of different individuals of the same species. Unlike the GFMT, facial images were captured in ambient environments and therefore contained natural variability in the appearance of the faces (see ref.^75^). We collected control data on this test from 48 participants in a voluntary online research registry (26 female, 22 male, mean age = 40, SD = 13).

*Fingerprint Matching Test.* This test, sourced from Tangen, et al.^76^, required participants to decide whether pairs of fingerprints originated from the same finger or two different fingers (see Fig. 6). Participants completed 60 trials (30 match, 30 non-match) which consisted of one ‘crime-scene’ print and one fully rolled ‘comparison’ print. Match pairs therefore contained natural variability due to the method of capture, while non-match pairs contained a non-matching but similar looking fingerprint selected by an automatic fingerprint identification system. We collected control data on this test from 1327 participants in a voluntary online research registry (899 female, 425 male, 3 non-binary, mean age = 44, SD = 13).

*Matching Familiar Figures Test (MFFT).* The MFFT^77^ measures cognitive style, impulsivity versus reflexivity (see Fig. 6). The test contains 20 trials where participants determine whether a target drawing is identical to one of the six variants shown in a gallery underneath, or if it is absent. We collected control data on this test from 1225 participants in a voluntary online research registry (833 female, 389 male, 3 non-binary, mean age = 45, SD = 12).

##### Professional face matching tests

*Expertise in Facial Comparison Test (EFCT).* The EFCT^40^ is a pairwise matching task with four components: the 2-s upright test, the 30-s upright test, the 2-s inverted test and the 30-s inverted test (see Fig. 1 for the upright conditions). The 2 and 30-s tests varied by exposure time but tested accuracy on the same image pairs. The upright and the inverted tests differed in the orientation of the images and each had different images pairs. For each trial, images remained visible for the prescribed exposure duration (2 or 30-s) and was either upright or inverted and then disappeared. Response options were as follows: (i) sure they are the same person; (ii) think they are the same person; (iii) do not know; (iv) think they are different people; and (v) sure they are different people. Participants could enter a response at any time during the image display or after the image pair disappeared. Each test consisted of 84 trials (half same identity, half different identity) and participants completed the 2-s test first and then immediately completed the 30-s test. Normative accuracy data from 32 controls and 27 forensic examiners was sourced from White, et al.^40^.

**Figure 7:**
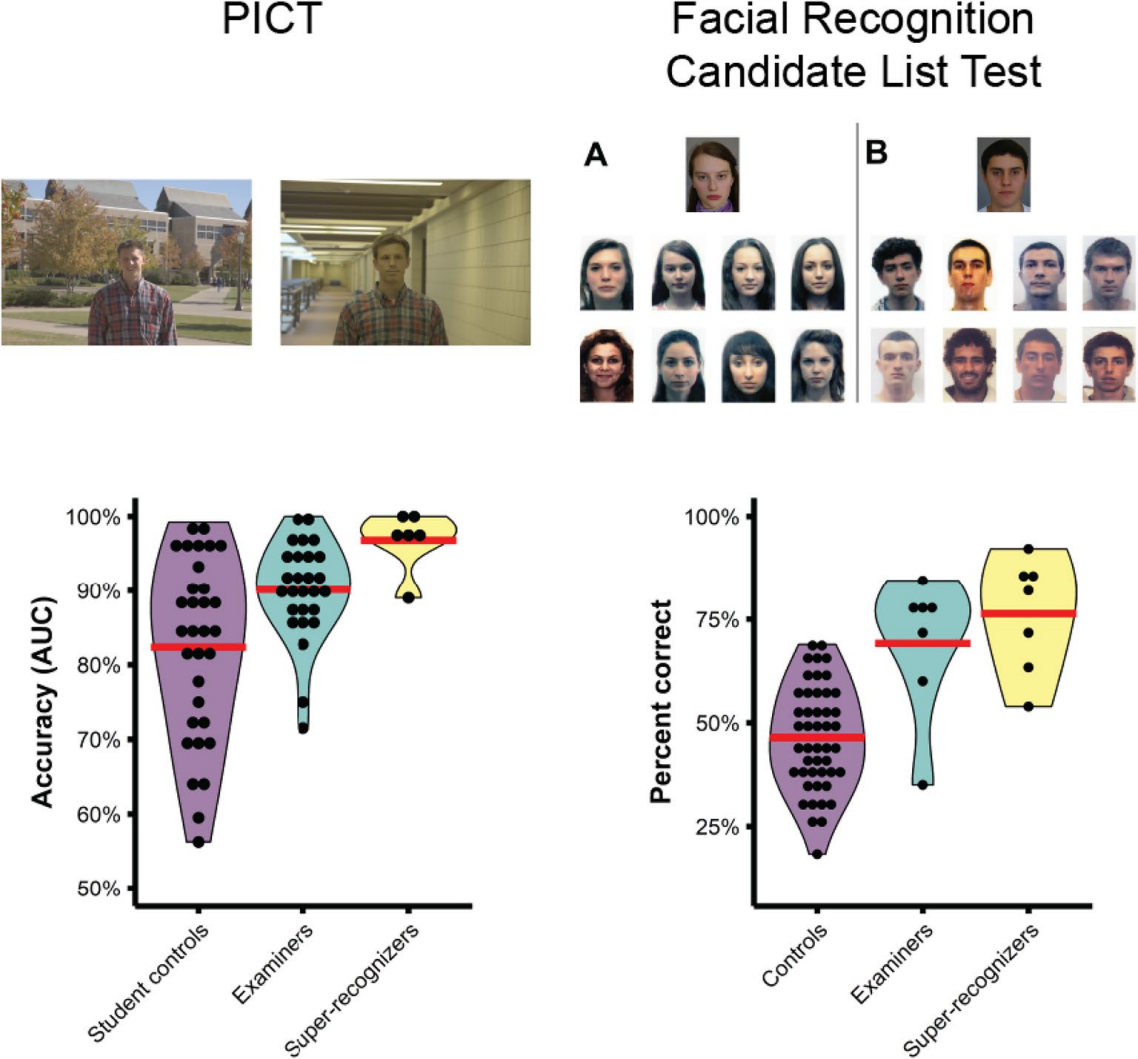
Super-recognizers’ and forensic examiners’ accuracy on professional face matching tasks. Violin plots show the distribution of performance for student controls, forensic examiners and super-recognizers. Red lines show group means. Super-recognizers outperformed controls on both tests but were statistically equivalent to forensic examiners. The top row shows example stimuli for the PICT^40^ and Facial Recognition Candidate List Test^41^. Facial Recognition Candidate List Test images are representative examples as the test stimuli are real passport images which we cannot show for privacy reasons.

*Person Identification Challenge Test (PICT).* The PICT^40^ uses images that contain both face and body information (see Fig. 7) and was created by selecting image pairs for which DNNs made 100% errors (see ref.^43^). Participants saw 40 pairs of images presented side by side and the response options were as follows: (i) sure they are the same person; (ii) think they are the same person; (iii) do not know; (iv) think they are different people; and (v) sure they are different people. Half the trials showed a match pair while the other half showed a non-match pair. Normative accuracy data from 32 controls and 27 forensic examiners was sourced from White, et al.^40^.

*Facial Recognition Candidate List Test.* The Facial Recognition Candidate List Test^41^ was designed to test performance of passport issuance officers on a task that modelled their daily work (see Fig. 7). This test used real passport facial images and required participants to determine whether a target ‘applicant’ was also one of the people shown in an eight-image gallery beneath it. ‘Foil’ images were selected by facial recognition software to be those most similar in appearance to the applicant image from the entire Australian passport image database. The target image when present in the gallery was taken on average 5 years prior to the applicant image for child and adolescent targets and on average 10 years prior for adult targets. Participants responded that the person was absent or by selecting the matching gallery image. A target was present on 50% of trials and when present was randomly allocated to one of the eight gallery positions. The test consists of 180 trials and the presentation of the stimulus array was limited to 18-s, after which participants were forced to make a response. Normative accuracy data from 47 controls and 7 forensic examiners was sourced from White, et al.^41^.

#### Procedure

Super-recognizers completed the CFMT + and the GFMT online prior to visiting to the lab. One super-recognizer (YS) also completed the UNSW Face Test at this time. Upon visiting the lab, the super-recognizers completed the CFMT-Aus, Models test, UNSW Face Test, EFCT, PICT and the Facial Recognition Candidate List Test in a randomised order. One super-recognizer (CT) was unable to complete all the tests during the lab visit. After visiting the lab, super-recognizers were sent a link to an online survey platform (Qualtrics; Provo, UT) to complete the Primate Face Matching Test, Fingerprint Matching Test and MFFT.

### International forensic proficiency test for face identification practitioners

Forensic proficiency tests are the standard method of assessing forensic practitioners’ abilities in the forensic sciences and are typically created by forensic organisations to be representative of forensic casework. They are intended to ensure practitioners are performing at an acceptable level and often form part of forensic accreditation requirements. The international forensic proficiency test reported here was administered by the European Network of Forensic Science Institutes’ Digital Image Working Group in 2018.

The proficiency test consisted of twenty 1-to-1 pairwise comparisons (13 match, 7 non-match). Subjects (14 male, 6 female) were photographed front-on using standard cameras, mobile phone cameras, webcams, or photobooths. Some photos were also sent via WhatsApp, interpolated, copy and pasted, or scanned from hardcopies (which can affect image resolution) to mimic real-world forensic casework. Environmental factors were not controlled, so facial expression, image quality, head angle, glasses, and hairstyle vary across the images. Nonetheless, all were front-facing images that were classified as being suitable for comparison by human experts. Participants were aware these image characteristics may be present in the images and were told the number of years between photos in each comparison (3 comparisons: 0 to 2 years, 13 comparisons: 2 to 8 years, 4 comparisons: 8 or more years).

Participants decided if each comparison showed the same person or different people using an 11-point response scale from − 5 (*Extremely strong support different people*) to 5 (*Extremely strong support same person*), where the midpoint 0 indicates the comparison provides “inconclusive” evidence one way or the other. Note that we have shortened these response scale labels for brevity (see supplementary materials for verbatim wording). Participants were also asked to indicate how long they had spent on the test.

Participants had 2 months to complete the test using their standard operating procedures. In some departments, practitioners ordinarily produce decisions on their own but in others, practitioners work together to produce decisions on behalf of a laboratory^65^. Procedures vary substantially between laboratories. Some will merely have a second practitioner check the spelling, grammar or terminology of a primary practitioner, whereas others will have one or more practitioners conduct a blind complete repeat analysis of the imagery, and everything in between^79^.

The proficiency test was completed by 16 *forensic examiners* and 19 *forensic laboratories* from 27 police, government, and private industry forensic departments in 14 countries in Europe, Africa, Oceania and the Middle East. Demographic information is unavailable for these participants. Forensic laboratories contained between 2 and 7 (mean 2.9) forensic examiners, facial reviewers, super-recognizers or other staff.

We recruited an additional 37 *super-recognizers* to complete the proficiency test. Seven were police officers working in a super-recognizer unit (M = 38 years, 5 male, 2 female). Six participated in the original lab-based benchmarking tests. The remaining super-recognizers were recruited from our international registry of civilian super-recognizers, created by screening the face recognition abilities of 1600 people. The 24 super-recognizers who had not participated in the original lab-based benchmarking tests were invited to participate if they scored 2SDs or more above the mean on the GFMT^38^, CFMT +^17^, and UNSW Face Test^74^. Demographic information is available for 18 of the 30 civilian super-recognizers, and indicates the super-recognizer sample is approximately 39% male and 61% female, with an approximate mean age of 35 years. Super-recognizers were not compensated for their time.

We also recruited 106 *novices* as a control group. Sixty-five were police officers from London’s Metropolitan Police Service with no face identification experience (M = 42 years, 45 male, 20 female). They were paid their normal wage. The remaining 41 novices were undergraduate psychology students from UNSW Sydney who participated in return for course credit. All novice participants completed the test online in a single testing session. Recruitment of additional human subjects was approved by the UNSW Human Research Ethics Advisory Panel and the Open University Human Research Ethics Committee, and informed consent was obtained. Finally, we selected the 10 open-source deep learning *face recognition DNNs* we had access to (see Table 1) to complete the test.

**Table 1:** The open-source facial recognition DNNs used in the international forensic proficiency test.

*Name*	*Model*	*Training*	*Python library*
*DNN1*	VGG16^80^	VGGFace^80^	Keras
*DNN2*	ResNet34^81^	VGGFace^80^; Face Scrub dataset^82^ and images from the internet^83^	Pytorch
*DNN3*	ResNet50^81^	VGGFace2^84^	Keras
*DNN4*	ResNet50^81^	VGGFace2^84^	Pytorch
*DNN5*	ResNet50^81^	MS-Celeb-1 M dataset^85^ fine-tuned on VGGFace2^84^	Pytorch
*DNN6*	Se-ResNet50^86^	VGGFace2^84^	Pytorch
*DNN7*	Se-ResNet50^86^	MS-Celeb-1 M dataset^85^ fine-tuned on VGGFace2^84^	Pytorch
*DNN8*	ArcFace^87^	CASIA^88^, VGGFace2^84^, MS1MV2^87^ and DeepGlint-Face^89^	DeepFace^90^
*DNN9*	Facenet^91^	VGGFace2^84^	DeepFace^90^
*DNN10*	Facenet512^91^	VGGFace2^84^	DeepFace^90^

To calculate fusion effects for each fusion group, we randomly sampled participants from each of the groups being fused and statistically averaged their judgments to form a single rating on each face pair. When fusing human and DNNs, DNN similarity scores were rescaled to the range of human ratings and combined with randomly sampled participants. The sampling procedure was repeated 1000 times for each fusion group and the combined ratings for each sample was used to calculate the Area Under the ROC Curve (AUC).

## Supplementary Information

Supplementary Information 1.

Supplementary Information 2.

## Data Availability

All data are available in supplementary materials.
