# Supplementary material for: Diverse types of expertise in facial recognition
Source: Sci Rep. 2023 Jul 14;13:11396. doi: 10.1038/s41598-023-28632-x (PMC10349110; doi:10.1038/s41598-023-28632-x)
Supplement: Supplementary file 1 — Supplementary Information 1. [file 41598_2023_28632_MOESM1_ESM.pdf]

SUPPLEMENTARY MATERIALS

Diverse types of expertise in facial recognition

Alice Towler[ab†*], James D. Dunn[a†], Sergio Castro Martínez[c], Reuben Moreton[d], Fredrick Eklöf[e], Arnout Ruifrok[f], Richard I. Kemp[a] & David White[a]

[a] School of Psychology, University of New South Wales, Sydney 2052, Australia; [b] School of Psychology, The University of Queensland, Brisbane 4072, Australia; [c] Sección Técnicas Identificativas, Comisaría General de Policía Científica, Madrid 28039, Spain; [d] School of Psychology, The Open University, Milton Keynes MK7 6AA, United Kingdom; [e] Forensic Imaging Biometrics, Information Technology Section, National Forensic Centre, Swedish Police Authority, Linköping 581 94, Sweden; [f] Forensic Biometrics, Netherlands Forensic Institute, The Hague 2497 GB, The Netherlands; [†]Joint 1st-author

Corresponding author: Dr Alice Towler, a.towler@uq.edu.au.

Benchmarking super-recognizers against forensic examiner and novice norms on lab-based tests

Individual-Level Analysis of Super-Recognizers vs. Norms on Lab-Based Tests

We observed some variability in the degree of superiority for each super-recognizer across the different tests (see Table S1). We assessed individual-level performance on each test using Crawford and Howell's modified one tailed t-test for single cases [1]. All super-recognizers satisfied the clinical criteria for abnormality on at least one of the lab tests. First on the face matching tests, 5 of the 7 super-recognizers' scored 1.96 SDs above the mean on the GFMT [2] (100% on the test), 1 scored 1.7SDs above the mean, and another 1 scored 1.4SDs above the mean. On the Models test [3], 6 of 7 super-recognizers scored above 1.7 SD.

For the face memory tests, 5 out of the 7 super-recognizers scored more than 2 standard deviations (SD) above the mean on the CFMT+ (i.e., > 93%)[4], and the remaining 2 scored 1.7SDs above the mean. Only 2 of the 7 scored above 1.7 SD on the CFMT-Aus [5], while 6 of 7 scored above 1.7 on the UNSW Face Test [6].

Table S1. Participant's accuracy on each of the tests in the lab-based assessment. Values in boldface indicate performance deviating more than 1.7 *SD*s from the mean of the normative data.

**Demographics**	*Subject*<br>*Age*<br>*Gender*	DP<br>31<br>M	TI<br>37<br>M	DB<br>27<br>F	HC<br>48<br>M	CM<br>24<br>F	YS<br>25<br>F	CT<br>46<br>M	**SR**<br>Mean<br>(SD)	**Normative**<br>Mean (SD)	Mean Difference	*t*<br>ratio	*p* value	Cohen's *d*
**Face Matching Tests**	*GFMT*	95	**100**	**100**	**100**	**98**	**100**	**100**	99 (2)	81 (10)	18	4.79	< .001	3.02
	Models	**94**	**98**	**98**	**96**	**96**	**98**	-	96 (1)	74 (11)	23	5.10	< .001	3.70
**Face Memory Tests**	*CFMT+*	**95**	**100**	**95**	**91**	**92**	**97**	**94**	95 (3)	69 (12)	26	5.60	< .001	3.41
	CFMT-Aus	93	**100**	**97**	96	93	95	94	96 (2)	80 (10)	15	3.97	< .001	2.43
**General Face Identification Test**	*UNSW FT*	63	**75**	**78**	**68**	**77**	**90**	**74**	75 (8)	59 (6)	16	7.18	< .001	2.28
**Object Matching Tests**	*Primates*	76	88	83	**92**	85	80	88	84 (5)	75 (8)	9	3.09	.003	1.47
	Fingerprints	92	**97**	72	87	80	82	83	85 (8)	77 (9)	7	2.08	.038	0.83
	MFFT	75	93	88	75	90	93	90	86 (8)	82 (11)	3	0.76	.448	0.34
**Professional Face Matching Tests**	*EFCT - 2s*	**94**	**97**	**97**	**93**	**95**	**99**	**95**	96 (2)	77 (9)	18	5.39	< .001	3.27
	EFCT - 30s	97	99	**100**	**100**	99	**100**	98	99 (1)	84 (9)	15	4.26	< .001	2.88
	PICT	97	100	100	98	89	97	-	97 (4)	82 (12)	15	2.88	.007	1.79
	FR CLT	54	**72**	**86**	63	**85**	**92**	**88**	77 (14)	46 (12)	31	5.98	< .001	2.29
**Face Inversion Effect**	*EFCT - 2s*	29	18	8	18	14	17	-	17 (7)	17 (9)	0	0.05	.964	0.02
	EFCT - 30s	16	7	3	18	7	7	-	10 (6)	15 (7)	-5	1.72	.095	-0.81

Extended Group-Level Analysis of Super-Recognizers vs. Norms on Lab-Based Tests

Super-recognizers' individual and group scores on each test are shown against the normative scores in the main manuscript Figure 7. For the face matching tests, super-recognizers outperformed the normative mean by 22% on the GFMT (99% vs. 81%; $t(199) = 4.79$, $p < .001$, Cohen's $d = 3.02$) and by 31% on the Models face matching test (96% vs. 74%; $t(58) = 5.10$, $p < .001$, Cohen's $d = 3.70$). Similarly on the face memory tests, super-recognizers outperformed the normative mean by 37% on the CFMT+ (95% vs. 69%; $t(259) = 5.60$, $p < .001$, Cohen's $d = 3.41$) and by 19% on the CFMT-Aus (96% vs. 80%; $t(80) = 3.97$, $p < .001$, Cohen's $d = 2.43$). Finally, super-recognizers outperformed the normative mean by 27% on the UNSW Face Test (75% vs. 59%; $t(295) = 7.18$, $p < .001$, Cohen's $d = 2.28$).

Super-recognizers also outperformed norms when matching non-human faces and other objects. Super-recognizers outperformed the norm on a Primate Face Matching Test by 13% (84% vs. 75%; $t(53) = 3.09$, $p = .003$, Cohen's $d = 1.47$) and a Fingerprint Matching Test by 9% (85% vs. 77%; $t(1332) = 2.08$, $p = .038$, Cohen's $d = 0.83$). Super-recognizers did not however, outperform the norm on the Matching Familiar Figures Test [7] (MFFT), scoring 4% higher than controls (86% vs. 82%; $t(1230) = 0.76$, $p = .448$, Cohen's $d = 0.34$). While super-recognizers showed an advantage on two of the three object matching tests, their advantage was much larger on the face identification tasks reported above (Mean face identification test: Cohen's $d = 2.97$ vs. Mean object matching test: Cohen's $d = 0.88$).

Extended EFCT 2sec vs. 30sec Analysis

Super-recognizers were more accurate than both student controls and forensic examiners when given only 2 seconds to view the faces (vs. Students, 2 seconds: 96% vs 77%; $t(35) = 10.17$, $p < .001$, Cohen's $d = 2.25$; vs. Students, 30 seconds: 99% vs 84%, $t(35) = 8.86$, $p < .001$, Cohen's $d = 1.78$; vs. Examiners, 2 seconds: 96% vs 81%; $t(32) = 4.69$, $p < .001$, Cohen's $d = 1.99$; vs. Examiners, 30 seconds: 99% vs 93%, $t(32) = 4.17$, $p < .001$, Cohen's $d = 1.77$). In contrast, White, et al. [8] found that forensic examiners only outperformed student controls on the EFCT when participants were given 30 seconds to view the images (93% vs. 84%; $t(57) = 4.92$, $p < .001$, Cohen's $d = 1.29$), and not when given 2 seconds (81% vs. 77%; $t(57) = 1.67$, $p = .099$, Cohen's $d = 0.44$). This finding shows that super-recognizers can achieve high levels of accuracy after only a short exposure, whereas forensic examiners' expertise takes longer and appears contingent on a slower method of comparison. This finding points to differences in the perceptual processes underlying the expertise of super-recognizers and forensic examiners.

International forensic proficiency test for face identification practitioners

Table S2. Errors at each point on the response scale for super-recognizers who completed the test with the raw image materials vs. those who completed the task online. Mann-Whitney U comparisons between errors made at each point on the response scale for the two groups. Significant comparisons are shaded in grey.

		-5	-4	-3	-2	-1	1	2	3	4	5
**Raw Mats. vs. Online**	U	145.50	154.50	160.00	132.00	150.50	158.50	117.00	142.00	156.00	144.00
	p	.339	.693	.931	.244	.545	.879	.089	.423	.810	.507

Table S3. Frequency of responses at each point on the response scale for super-recognizers who completed the test with raw image materials vs. those who completed the task online. Mann-Whitney U comparisons between the frequency with which each group used each point on the response scale. Significant comparisons are shaded in grey.

		-5	-4	-3	-2	-1	0	1	2	3	4	5
**Raw Mats. vs. Online**	U	163.00	151.00	117.50	110.00	151.00	170.50	165.50	89.00	170.50	143.00	145.00
	p	.803	.528	.089	.049	.462	.969	.846	.009	.988	.390	.427

Table S4. International Forensic Proficiency Test Response Scale

Response	Values* of likelihood ratio	Response scale labels
**+5**	1,000,000 and above	The observations provide **extremely strong support** to the proposition that it is the same person relative to the proposition that it are different persons.
**+4**	10,000-1,000,000	The observations provide **very strong support** to the proposition that it is the same person relative to the proposition that it are different persons.
**+3**	100-10,000	The observations provide **strong support** to the proposition that it is the same person relative to the proposition that it are different persons.
**+2**	10-100	The observations provide **support** to the proposition that it is the same person relative to the proposition that it are different persons.
**+1**	2-10	The observations provide **weak support** to the proposition that it is the same person relative to the proposition that it are different persons.
**0**	1-2	The observations **support neither** the proposition that it is the same person **nor** the proposition that it are different persons.
**-1**		The observations provide **weak support** to the proposition that it are **not** the same persons relative to the proposition that it is the same person.
**-2**		The observations provide **support** to the proposition that it are **not** the same persons relative to the proposition that it is the same person.
**-3**		The observations provide **strong support** to the proposition that it are **not** the same persons relative to the proposition that it is the same person.
**-4**		The observations provide **very strong support** to the proposition that it are **not** the same persons relative to the proposition that it is the same person.
**-5**		The observations provide **extremely strong support** to the proposition that it are **not** the same persons relative to the proposition that it is the same person.

* Likelihood ratios corresponding to the inverse (1/X) of these values (X) will express the degree of support for the specified alternative compared to the first proposition.

Extended AUC analyses

Performance on the proficiency test was compared using Area Under the ROC Curve (AUC) in a one-way ANOVA with Group (novices, super-recognizers, forensic examiners, DNNs, laboratories) as the between subjects factor. There was a significant effect of Group ($F(4,183) = 26.5$, $p < .001$, $\eta_p^2 = .37$) which we followed up with planned comparisons. All groups had significantly higher AUC than novices (novices vs. forensic examiners: $t(120) = 4.79$, $p < .001$, Cohen's $d = 1.29$; novices vs. super-recognizers: $t(141) = 5.50$, $p < .001$, Cohen's $d = 1.05$; novices vs. DNNs: $t(114) = 2.98$, $p = .004$, Cohen's $d = 0.99$; novices vs laboratories: $t(123) = 7.65$, $p < .001$, Cohen's $d = 1.91$). However, there were no significant differences in AUC between super-recognizers, forensic examiners or DNNs (super-recognizers vs. forensic examiners: $t(51) = 1.21$, $p = .232$, Cohen's $d = 0.36$; super-recognizers vs. DNNs: $t(45) = 0.13$, $p = .900$, Cohen's $d = 0.04$; forensic examiners vs. DNNs: $t(24) = 1.29$, $p = .210$, Cohen's $d = 0.52$). Finally, forensic laboratories outperformed all expert groups (laboratories vs. super-recognizers: $t(54) = 4.60$, $p < .001$, Cohen's $d = 1.30$; laboratories vs. forensic examiners: $t(33) = 3.68$, $p < .001$, Cohen's $d = 1.25$; laboratories vs. DNNs: $t(27) = 6.37$, $p < .001$, Cohen's $d = 2.49$).

Completion time analyses

Test completion times are shown in Figure S2. No test time completion data was recorded for one forensic examiner and one forensic laboratory so they were excluded from this analysis. Test completion times for participants who completed the test online were recorded by the testing software (18 super-recognizers, 65 novices). Test completion times were estimated by the remaining participants who completed the test using the raw images and static response document (19 super-recognizers, 15 forensic examiners, 41 novices, 18 forensic laboratories).

To assess the equivalence of measured and estimated completion times, we conducted an independent-samples t-tests between the measured and estimated completion times of super-recognizers and novices. These tests revealed no significant difference between measured and estimated completion times for super-recognizers [$t(35) = 1.29$, $p = .207$] or novices [$t(104) = .70$, $p = .488$].

Test completion time was analysed in a one-way ANOVA with Group (novices, super-recognizers, forensic examiners, DNNs, forensic laboratories) as the between subjects factor. There was a significant effect of Group ($F(4,181) = 30.2$, $p < .001$, $\eta_p^2 = .40$) which we followed up with planned comparisons. Forensic laboratories took significantly longer to complete the test than novices, super-recognizers, and DNNs (laboratories vs. novices: $t(122) = 9.62$, $p < .001$, Cohen's $d = 2.45$; Laboratories vs. super-recognizers: $t(53) = 5.38$, $p < .001$, Cohen's $d = 1.55$; laboratories vs. DNNs: $t(26) = 2.96$, $p = .006$, Cohen's $d = 1.17$) but were not statistically different to forensic examiners (laboratories vs. forensic examiners: $t(31) = 1.39$, $p = .175$, Cohen's $d = 0.49$). Forensic examiners also took significantly longer than novices, super-recognizers and DNNs (forensic examiners vs. novices: $t(119) = 7.92$, $p < .001$, Cohen's $d = 2.18$; forensic examiners vs. super-recognizers: $t(50) = 4.26$, $p < .001$, Cohen's $d = 1.30$; forensic examiners vs. DNNs: $t(23) = 2.50$, $p = .020$, Cohen's $d = 1.02$). Finally, super-recognizers took longer than novices and DNNs (super-recognizers vs. novices: $t(141) = 3.93$, $p < .001$, Cohen's $d = 0.75$; super-recognizers vs. DNNs: $t(45) = 2.73$, $p = .009$, Cohen's $d = 0.97$) but novices were not significantly different to DNNs ($t(114) = 1.77$, $p = .079$, Cohen's $d = 0.59$).

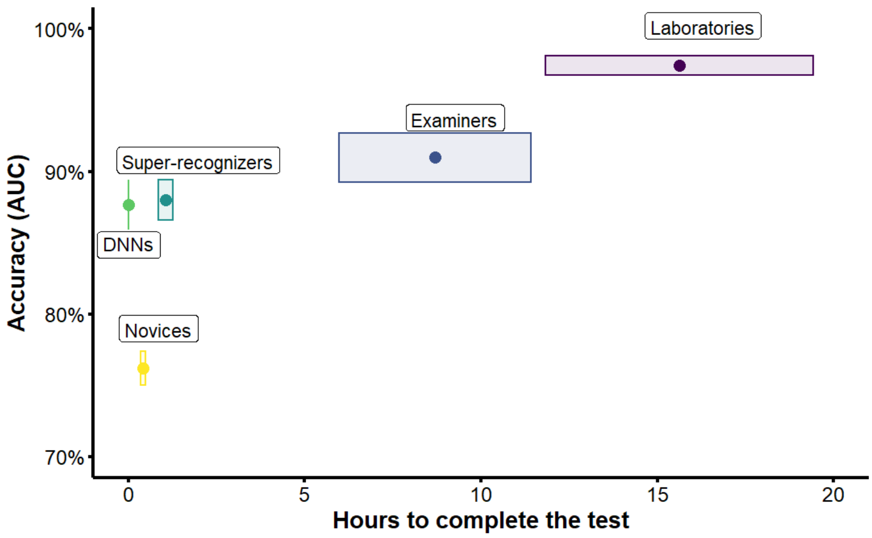

Fig. S2. Variability in time to complete the proficiency test. *Markers show the mean hours to complete the test for each group against their accuracy (AUC). The shaded area around each marker shows the standard error on each measure.*

Extended version of Figure 3

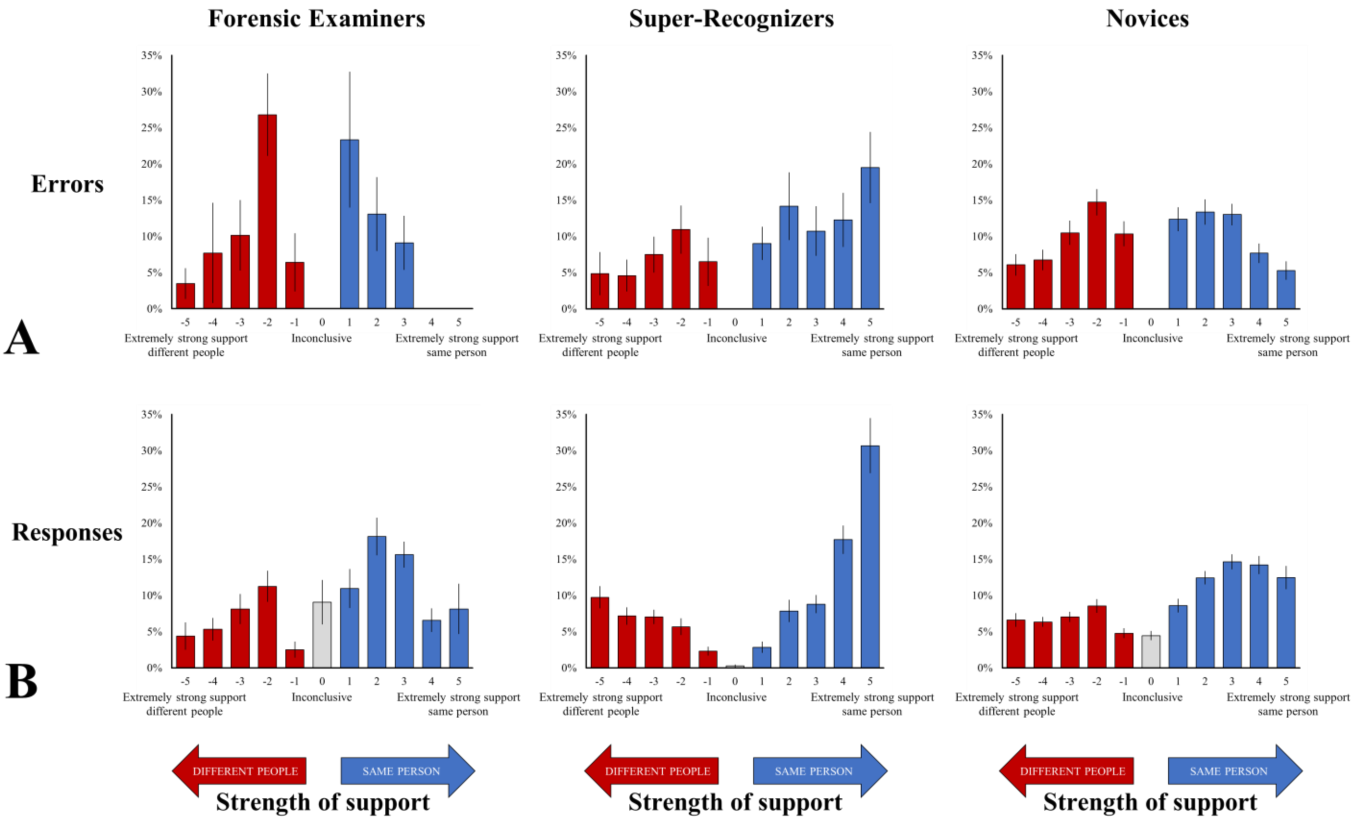

Fig. S3. Extended version of Figure 3 - Error and response distributions across the 11-point response scale for forensic examiners, super-recognizers, and novices. *Error bars show standard error of the mean.*

Table S5. Errors at each point on the response scale. Mann-Whitney U comparisons between groups of the errors made at each point on the response scale. Significant comparisons are shaded in grey. SR = super-recognizer, Ex = forensic examiner, Nov = novices.

		-5	-4	-3	-2	-1	1	2	3	4	5
**SR v Ex**	U	225.5	222.0	218.5	135.0	230.0	189.5	219.5	229.5	169.0	149.5
	p	.735	.633	.642	.012	.882	.194	.699	.898	.037	.014
**SR v Nov**	U	1769.0	1724.5	1699.5	1616.5	1559.0	1542.0	1686.0	1577.5	1832.0	1477.5
	p	.318	.234	.239	.130	.041	.051	.250	.087	.653	.005
**Ex v Nov**	U	662.5	586.0	660.5	470.0	579.0	661.5	658.0	586.5	494.0	572.0
	p	.739	.232	.776	.046	.257	.797	.774	.344	.029	.110

Table S6. Frequency of responses at each point on the response scale. Mann-Whitney U comparisons between groups of the frequency with which they use each point on the response scale. Significant comparisons are shaded in grey. SR = super-recognizer, Ex = forensic examiner, Con = Control.

		-5	-4	-3	-2	-1	0	1	2	3	4	5
**SR v Ex**	U	177.0	253.5	290.5	173.0	295.5	157.0	152.5	129.5	155.5	124.5	114.5
	p	.017	.392	.911	.013	.991	.000	.002	.001	.006	.001	.000
**SR v Con**	U	1407.5	1836.5	1857.5	1635.5	1561.0	1191.0	1246.0	1353.5	1303.0	1581.0	1058.0
	p	.007	.550	.621	.120	.042	.000	.001	.004	.002	.077	.000
**Ex v Con**	U	712.5	777.5	784.5	648.0	678.5	709.0	738.5	574.5	746.5	550.0	670.0
	p	.266	.576	.618	.120	.160	.244	.392	.035	.435	.022	.155

Correct, Incorrect & Inconclusive decisions of forensic examiners and super-recognizers

To explore the decisional strategies of forensic examiners and super-recognizers in greater detail, we examined their proportion of correct, incorrect and inconclusive responses, by categorising responses of -1 to -5 as "same person" decisions and responses of 1 to 5 as "different person" decisions.

We found that super-recognizers and forensic examiners used the response scale differently (see Figure 3A). While super-recognizers and examiners made similar proportions of correct (83.6% vs. 79.4%) and incorrect (16.1% vs. 11.6%) decisions, forensic examiners responded "inconclusive" (9.1%) far more often than super-recognizers, who almost never responded "inconclusive" (0.3%) [correct: $t(51) = 1.63$, $p = .110$, Cohen's $d = 0.48$; incorrect: $t(51) = 1.75$, $p = .086$, Cohen's $d = 0.53$; inconclusive: $t(51) = 4.37$, $p < .0001$, Cohen's $d = 1.01$]. In fact, 0.3% of super-recognizers' responses represents just two decisions by two different super-recognizers across the entire test.

This stark difference in "inconclusive" responses suggests that forensic examiners deliberately avoid making identification decisions in some instances. The authors note that in high-stakes real-world forensic practice, forensic examiners routinely declare some comparisons "inconclusive" to avoid making errors that could have profound life-changing consequences for the people involved, especially when the comparison involves poor-quality imagery or large age differences. Indeed, Norell, et al. [9] observed that forensic examiners were more likely to respond "inconclusive" as image quality decreased. However, there it was unclear if forensic examiners' increased tendency to respond "inconclusive" reflected an underlying sensitivity to which cases are more likely to result in errors (i.e. a strategic conservatism), or whether it simply reflected a generalised conservatism applied uniformly across comparisons that reduces the number of errors *and* correct decisions to a similar extent.

To investigate this question, we compared the proportion of forensic examiners who declared each of the 20 comparisons as "inconclusive" to super-recognizers' accuracy on those comparisons. We found a strong negative correlation (Spearman's $\rho = -.68$, $p = .001$), such that the more a comparison was declared "inconclusive" by forensic examiners, the worse super-recognizers performed. And, considering only comparisons where participants made a "same person" or "different people" decision i.e. did not respond

“inconclusive”; see [10], forensic examiners were slightly *more accurate* [87.9% vs. 83.9%; $t(51) = 1.57$, $p = .122$, Cohen’s $d = 0.47$] and made *fewer errors* than super-recognizers [12.1% vs. 16.1%; $t(51) = 1.55$, $p = .127$, Cohen’s $d = 0.46$]. Together, this evidence indicates that forensic examiners are sensitive to which comparisons have a greater chance of error, and thus strategically choose *not* to make an identification decision in those cases. This strategy appears to help forensic examiners avoid the errors made by super-recognizers.

Sensitivity & Criterion

We calculated sensitivity using d′ (see Figure S4). We conducted a one-way ANOVA on the sensitivity data with Group (super-recognizers, forensic examiners, novices) as the between-subjects factor. There was a significant effect of Group [$F(4, 177) = 24.03$, $p < .001$, $\eta_p^2 = .35$] which we followed up with planned comparisons. Super-recognizers and forensic examiners showed equivalent sensitivity [$t(51) = 1.53$, $p = .133$, Cohen’s $d = 0.46$]. Novices showed significantly lower levels of sensitivity than both super-recognizers [$t(141) = 5.61$, $p < .001$, Cohen’s $d = 1.07$] and forensic examiners [$t(120) = 6.14$, $p < .001$, Cohen’s $d = 1.65$].

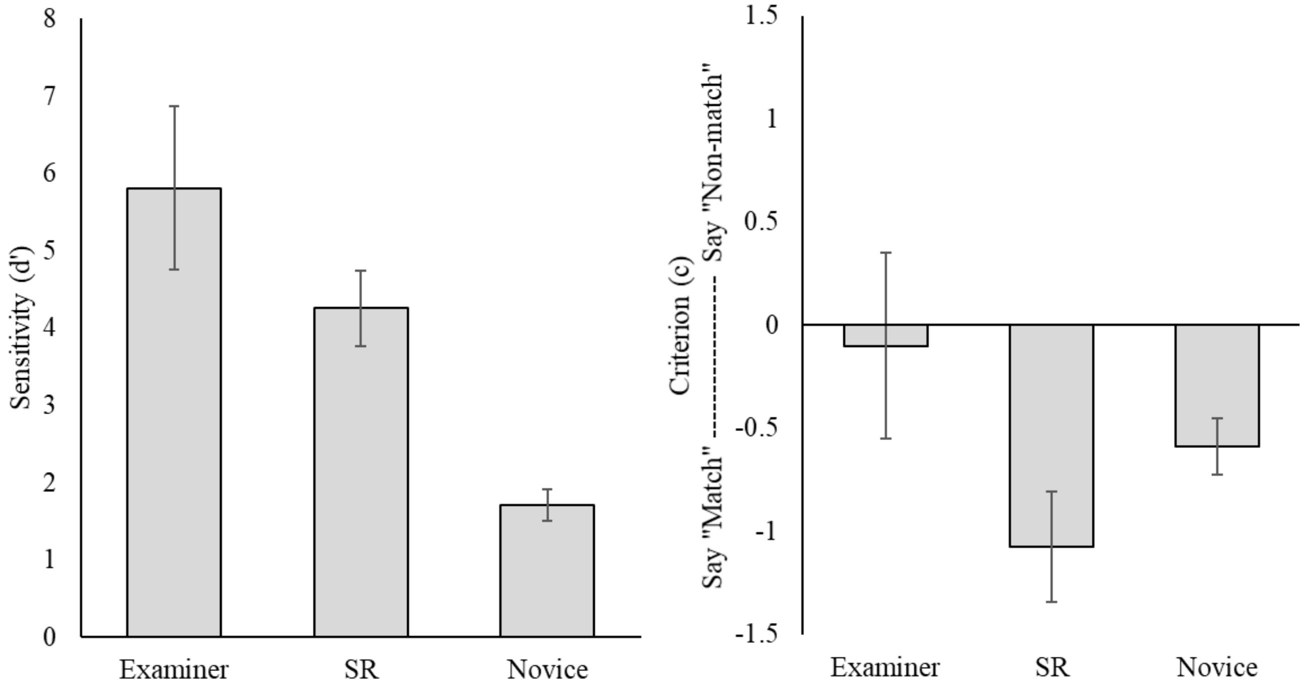

Fig. S4. Sensitivity and criterion for forensic examiners, super-recognizers, and novices. *Error bars show standard error of the mean.*

To calculate criterion we classified responses of 1 to 5 as “match” responses, and responses of -5 to -1 as “non-match” responses (see Figure S4). Responses of 0 were excluded from analysis of criterion. We conducted a one-way ANOVA on the criterion data with Group (super-recognizers, forensic examiners, novices) as the between-subjects factor. There was a significant effect of Group [$F(4, 177) = 3.72$, $p = .006$, $\eta_p^2 = .08$] which we followed up with planned comparisons. Super-recognizers showed a marginally significant stronger response bias than forensic examiners [$t(51) = 1.93$, $p = .059$, Cohen’s $d = 0.58$] and novices [$t(141) = 1.73$, $p = .086$, Cohen’s $d = 0.33$]. Forensic examiners showed an equivalent response bias to novices [$t(120) = 1.24$, $p = .216$, Cohen’s $d = 0.33$].

We also compared each groups’ criterion values to 0, which indicates a neutral response bias, using one-sample t-tests. Super-recognizers ($t(36) = 3.98$, $p < .001$, Cohen’s $d = 0.65$) and novices ($t(105) = 4.29$, $p < .001$, Cohen’s $d = 0.42$) had a significant response bias to say “same person”. Forensic examiners did not show a significant response bias ($t(15) = 0.22$, $p = .828$, Cohen’s $d = 0.06$).

We expected police super-recognizers might show a less pronounced response bias to say “same person” given their awareness of the serious real-world consequences of misidentifications. However, the same-person bias

was much stronger for police super-recognizers (M = -2.11) than for civilian super-recognizers [M = -0.83; $t(35)$ = 1.93, p = .062, Cohen's d = 0.82].

Agreement of facial similarity judgements

DNNs showed a high level of agreement with other DNNs for both "same person" pairs (average ρ = 0.53) and "different person" pairs (average ρ = 0.65), as indicated by the cluster of red pixels in the top right-hand corners of Figures 5A and 5B. Similarly, humans tended to agree with each other for "same person" pairs (average ρ = 0.18) and "different person" pairs (average ρ = 0.29), indicating that despite differences in how humans arrive at their judgments they converge on relatively similar assessments of facial similarity.

For "same person" pairs (Figure 5A), forensic examiners (average ρ = 0.42) and forensic laboratories (average ρ = 0.44) show higher levels of agreement within their groups than super-recognizers (average ρ = 0.18) and novices do (average ρ = 0.15). For "different person" pairs (see Figure 5B), forensic examiners (average ρ = 0.50), forensic laboratories (average ρ = 0.42), super-recognizers (average ρ = 0.44) and novices (average ρ = 0.23) show similar levels of agreement within their groups. The high level of agreement among forensic examiners and forensic laboratories for "same person" pairs may be a consequence of forensic practitioners' training to 'harmonise' their responses, i.e. for different practitioners examining the same image pair to arrive at the same point on the response scale. Greater agreement in responses across members of professional groups is often taken to indicate greater objectivity of forensic face identification methods, and so is perceived as desirable by the forensic science community [11-16].

Fusion analyses

We examined the benefits of fusing decisions from small groups of face identification experts. To do this, we randomly sampled sets of responses made by groups of 2 and 3 individuals 1000 times, computed average responses of each set to each image pair, and then calculated the accuracy of the collective decisions made by the set using AUC (see main text Figure 6). To analyse whether the fused responses improved accuracy, we performed planned Wilcoxon rank sum one-tailed tests predicting that each level of fusion groups would be more accurate than the smaller fusion or individual response counterparts.

Replicating previous work [17-19], we find that all fusion pairs (i.e., 2 x novices, 2 x super-recognizers, 2 x forensic examiners) showed significant improvements in accuracy relative to individual decisions from the same group (novices: W = 35970, p < .001; super-recognizers: W = 11758, p < .001; forensic examiners: W = 5772, p = .028). The best fusion results however were achieved from fusion human decision makers with DNNs. Table S10 shows the median and deviation in AUC achieved with fusion of each DNN with examiners and super-recognizers. We also find that all fusion triplets (i.e., 3 x novices, 3 x super-recognizers, 3 x forensic examiners) showed significant improvements in accuracy compared to the fusion pair counterparts (novices: W = 416052, p < .001; super-recognizers: W = 403195, p < .001; forensic examiners: W = 424256, p < .001).

Table S10. Median and SD of AUC achieved by fusing each DNN with a single examiner, a single super-recognizer, or both an examiner and super-recognizer.

	Examiners		*Super-recognizers*		*Examiners + Super-recognizers*	
AUC	*Median*	*SD*	*Median*	*SD*	*Median*	*SD*
DNN1	0.969	0.033	0.964	0.038	0.981	0.024
DNN2	0.954	0.045	0.958	0.034	0.974	0.027
DNN3	0.963	0.037	0.970	0.030	0.981	0.024
DNN4	0.963	0.033	0.961	0.036	0.976	0.024
DNN5	0.923	0.043	0.944	0.041	0.964	0.032
DNN6	0.946	0.045	0.955	0.043	0.977	0.026
DNN7	0.943	0.035	0.944	0.048	0.974	0.028
DNN8	0.921	0.042	0.948	0.040	0.966	0.036
DNN9	0.964	0.042	0.962	0.034	0.977	0.026
DNN10	0.959	0.039	0.969	0.029	0.977	0.027

Further analysis of DNN fusions show combination of DNNs that produced the highest overall AUC (Table S10). Examination of the gains in performance from DNN fusion (Table S11) and correlation in similarity scores for image pairs (Table S12) suggests that gains from fusion is predicted by weaker correlations in similarity ratings. This relationship is confirmed by a significant negative correlation between system gains in AUC and correlation in similarity scores, $r(45) = -0.42$, $p = .004$.

Table S11. System AUC resulting from fusing pairs of DNN. **Bold** values show the standalone DNN AUC.

AUC	*DNN1*	*DNN2*	*DNN3*	*DNN4*	*DNN5*	*DNN6*	*DNN7*	*DNN8*	*DNN9*	*DNN10*
DNN1	**0.907**	0.912	0.912	0.923	0.868	0.890	0.890	0.923	0.923	0.945
DNN2		**0.912**	0.912	0.901	0.879	0.923	0.901	0.923	0.923	0.923
DNN3			**0.912**	0.923	0.901	0.934	0.901	0.945	0.923	0.945
DNN4				**0.901**	0.868	0.868	0.879	0.934	0.901	0.923
DNN5					**0.780**	0.857	0.846	0.879	0.857	0.890
DNN6						**0.802**	0.857	0.879	0.890	0.934
DNN7							**0.846**	0.923	0.890	0.901
DNN8								**0.868**	0.934	0.890
DNN9									**0.879**	0.912
DNN10										**0.956**

Table S12. Relative system gain in AUC from fusion compared to the better of the two standalone DNN AUC scores.

AUC	*DNN2*	*DNN3*	*DNN4*	*DNN5*	*DNN6*	*DNN7*	*DNN8*	*DNN9*	*DNN10*
DNN1	0.000	0.000	0.016	-0.038	-0.016	-0.016	0.016	0.016	-0.011
DNN2		0.000	-0.011	-0.033	0.011	-0.011	0.011	0.011	-0.033
DNN3			0.011	-0.011	0.022	-0.011	0.033	0.011	-0.011
DNN4				-0.033	-0.033	-0.022	0.033	0.000	-0.033
DNN5					0.055	0.000	0.011	-0.022	-0.066
DNN6						0.011	0.011	0.011	-0.022
DNN7							0.055	0.011	-0.055
DNN8								0.055	-0.066
DNN9									-0.044

Table S13. Pearson correlation's showing the association between each DNN's similarity ratings of the image pairs

AUC	*DNN2*	*DNN3*	*DNN4*	*DNN5*	*DNN6*	*DNN7*	*DNN8*	*DNN9*	*DNN10*
DNN1	0.892	0.894	0.758	0.769	0.789	0.760	0.610	0.786	0.892
DNN2		0.903	0.863	0.806	0.878	0.767	0.635	0.830	0.845
DNN3			0.824	0.865	0.815	0.789	0.675	0.802	0.900
DNN4				0.829	0.867	0.770	0.567	0.854	0.785
DNN5					0.858	0.778	0.575	0.765	0.869
DNN6						0.849	0.533	0.806	0.827
DNN7							0.368	0.781	0.750
DNN8								0.618	0.735
DNN9									0.856

Table S14. Median AUCs for individuals, and fused pairs and triplets (data plotted in Figure 6 of the manuscript)

AUC	*Human only or DNN only*	*Human + DNN*
Individuals		
SRs	0.879	
Examiners	0.912	
DNN10	0.956	
Pairs		
DNN10+DNN3	0.945	
SR+SR	0.945	
EX+EX	0.951	
EX+SR	0.951	
SR+DNN10		0.967
EX+DNN10		0.967
Triplets		
DNN10+DNN3+DNN1	0.945	
EX+EX+EX	0.956	
SR+SR+SR	0.962	
EX+EX+SR	0.967	
EX+SR+SR	0.967	
EX+EX+DNN10		0.978
SR+SR+DNN10		0.989
EX+SR+DNN10		0.989

References

1 Crawford, J. R. & Howell, D. C. Comparing an Individual's Test Score Against Norms Derived from Small Samples. *The Clinical Neuropsychologist* **12**, 482-486, doi:10.1076/clin.12.4.482.7241 (1998).

2 Burton, A. M., White, D. & McNeill, A. The Glasgow Face Matching Test. *Behavior Research Methods* **42**, 286-291, doi:10.3758/BRM.42.1.286 (2010).

3 Dowsett, A. J. & Burton, A. M. Unfamiliar face matching: Pairs out-perform individuals and provide a route to training. *British Journal of Psychology* **106**, 433-445, doi:10.1111/bjop.12103 (2014).

4 Bobak, A. K., Pampoulov, P. & Bate, S. Detecting superior face recognition skills in a large sample of young British adults. *Frontiers in Psychology* **7** (2016).

5 McKone, E. *et al.* Face ethnicity and measurement reliability affect face recognition performance in developmental prosopagnosia: Evidence from the Cambridge Face Memory Test–Australian. *Cognitive Neuropsychology* **28**, 109-146 (2011).

6 Dunn, J. D., Summersby, S., Towler, A., Davis, J. P. & White, D. UNSW Face Test: A screening test for super-recognisers. *PLoS ONE* **15**, e0241747 (2020).
7 Kagan, J. Reflection-impulsivity and reading ability in primary grade children. *Child Development* **36**, 609-628 (1965).
8 White, D., Phillips, P. J., Hahn, C. A., Hill, M. & O'Toole, A. J. Perceptual expertise in forensic facial image comparison. *Proceedings of the Royal Society of London B: Biological Sciences* **282**, 1814-1822, doi:10.1098/rspb.2015.1292 (2015).
9 Norell, K. *et al.* The effect of image quality and forensic expertise in facial image comparisons. *Journal of Forensic Sciences* **60**, 331-340, doi:10.1111/1556-4029.12660 (2015).
10 Towler, A. *et al.* Are forensic scientists experts? *Journal of Applied Research in Memory and Cognition* **7**, 199-208 (2018).
11 Edmond, G. *et al.* How to cross-examine forensic scientists: A guide for lawyers. *Australian Bar Review* **39**, 174-197 (2014).
12 Dror, I. E. *et al.* Cognitive issues in fingerprint analysis: Inter- and intra-expert consistency and the effect of a 'target' comparison. *Forensic Science International* **208**, 10-17 (2011).
13 Ulery, B. T., Hicklin, R. A., Buscaglia, J. & Roberts, M. A. Repeatability and reproducibility of decisions by latent fingerprint examiners. *PLoS ONE* **7**, 1-12, doi:10.1371/journal.pone.0032800 (2012).
14 Hicklin, R. A. *et al.* Accuracy and reproducibility of conclusions by forensic bloodstain pattern analysts. *Forensic Science International* **325**, 110856, doi:https://doi.org/10.1016/j.forsciint.2021.110856 (2021).
15 National Research Council. Strengthening forensic science in the United States: A path forward. (2009).
16 Rairden, A., Garrett, B. L., Kelley, S., Murrie, D. & Castillo, A. Resolving latent conflict: What happens when latent print examiners enter the cage? *Forensic Science International* **289**, 215-222, doi:https://doi.org/10.1016/j.forsciint.2018.04.040 (2018).
17 Jeckeln, G., Hahn, C. A., Noyes, E., Cavazos, J. G. & O'Toole, A. J. Wisdom of the social versus non-social crowd in face identification. *British Journal of Psychology* **109**, 724-735, doi:10.1111/bjop.12291 (2018).
18 Phillips, P. J. *et al.* Face recognition accuracy in forensic examiners, super-recognisers and algorithms. *Proceedings of the National Academy of Sciences* **115**, doi:10.1073/pnas.1721355115 (2018).
19 White, D., Burton, A. M., Kemp, R. I. & Jenkins, R. Crowd effects in unfamiliar face matching. *Applied Cognitive Psychology* **27**, 769-777 (2013).
